# Dynamics of brain-muscle interaction with neuromuscular fatigability: systematic review

**DOI:** 10.3389/fphys.2026.1719722

**Published:** 2026-04-20

**Authors:** Sarah Al Omari, Charlotte Moeyersons, Arne Defour, David Haslacher, Bart Jansen, David Beckwée, Eva Swinnen, Mahyar Firouzi

**Affiliations:** 1 Rehabilitation Research Group (RERE), Department of Physiotherapy, Human Physiology and Anatomy, Vrije Universiteit Brussel, Brussels, Belgium; 2 Department of Rehabilitation Sciences, Ghent University, Ghent, Belgium; 3 Department of Human Movement Sciences, Faculty of Behavioural and Movement Sciences, Vrije Universiteit Amsterdam, Amsterdam, Netherlands; 4 Department of Psychiatry and Neurosciences, Charité Universitätsmedizin Berlin, Corporate Member of Freie Universität Berlin, Humboldt-Universität zu Berlin, Berlin, Germany; 5 Department of Electronics and Informatics (ETRO), Engineering Sciences, Vrije Universiteit Brussel, Brussels, Belgium; 6 Interuniversity Microelectronics Centre (IMEC), Leuven, Belgium; 7 Department of Rehabilitation Sciences and Physiotherapy, University of Antwerp, Antwerp, Belgium; 8 Brain, Body and Cognition Research Group (BBCO), Faculty of Psychology and Educational Sciences, Vrije Universiteit Brussel, Brussels, Belgium

**Keywords:** corticomuscular coherence, electroencephalography, electromyography, fatigability, neurophysiology

## Abstract

**Introduction:**

Neuromuscular fatigability impairs motor performance in both healthy and neurological populations. Corticomuscular coherence (CMC), derived from EEG and EMG recordings, reflects the brain-muscle interaction during movement. However, the impact of neuromuscular fatigability on CMC in healthy and neurological populations remains unclear.

**Methods:**

A systematic search of PubMed, Web of Science, and Embase was conducted up to 02/02/2026. Eligible studies investigated CMC changes related to fatiguing tasks in healthy or neurological participants. Two reviewers independently screened, extracted data, and assessed the risk of bias.

**Results:**

Fifteen non-randomized experimental studies were included, comprising predominantly neurologically healthy adults (n= 174) and a limited number of individuals with neurological conditions (n= 14). Fatiguing tasks varied widely in muscle group, contraction type, mode, and intensity. Across studies, neuromuscular fatigability was associated with heterogeneous changes in CMC, most commonly involving reductions in beta band coherence as fatigue progressed. However, preserved or increased beta band CMC was also reported in both upper- and lower-limb tasks, particularly during sustained or low- to moderate-intensity contractions. Alpha and gamma band CMC were less reported across the included studies. No consistent or limb-specific pattern of CMC modulation emerged, with observed responses depending on task demands, contraction intensity, muscle group, and stage of fatigue. Evidence from neurological populations was sparse but suggested generally lower CMC magnitude and greater disruption during fatiguing tasks compared with healthy controls.

**Discussion:**

These findings indicate that fatigue-related changes in CMC do not reflect a uniform loss of corticomuscular coupling but rather task- and context-dependent adaptations in brain–muscle communication. Reductions in CMC may reflect diminished efficacy of corticospinal synchronization, whereas preserved or increased coherence may represent stabilization to maintain motor output with fatigue. By synthesizing how neuromuscular fatigability reshapes CMC across different experimental contexts and highlighting key methodological limitations, this review provides a framework to inform the design of future rehabilitation or neuromodulation trials targeting fatigability in both healthy and neurological populations.

## 1 Introduction

Fatigue is a common phenomenon that can impair motor performance in both healthy and clinical populations. It is generally classified into state fatigue, which refers to the temporary sensation of tiredness arising from activity or exertion, and trait fatigue, a more persistent, chronic feature (Van Geel et al., 2020). State fatigue can be quantified through performance decline and is often described as fatigability, which encompasses both cognitive and motor domains (Kratz et al., 2019). Within motor fatigability, neuromuscular fatigability specifically refers to the progressive loss of force-generating capacity due to impairments in neural drive and/or muscle function (Kratz et al., 2019). This phenomenon is commonly divided into central (often referred to as “neural”) and peripheral (or “muscular”) components, both of which significantly impair motor function and rehabilitation outcomes (Carroll et al., 2017; Welch and Kutlubaev, 2020; Juárez-Belaúnde et al., 2021; Royer et al., 2022).

Neuromuscular fatigability can impair the performance of essential activities of daily living, even in healthy individuals. Studies have shown that following fatiguing exercises of the hips and/or knees, individuals exhibit greater sway variability while standing, indicating diminished postural control and increased fall risk (Gribble and Hertel, 2004; Vuillerme et al., 2006; Salavati et al., 2007; Bizid et al., 2009). Beyond balance, fatigue disrupts fundamental aspects of motor control, such as muscle coordination and timing (Wilder et al., 1996; Corcos et al., 2002), slows reaction time (Lorist et al., 2002), and reduces movement accuracy (Jaric et al., 1999). For instance, during repetitive elbow flexion-extension tasks, fatigue of the extensors leads to consistent undershooting of the target position (Jaric et al., 1999). These fatigue-induced alterations in muscle performance and coordination disrupt task execution, contributing to larger movement errors, impaired sensorimotor integration, and hindered motor adaptation (Gates and Dingwell, 2008; Pethick and Tallent, 2022; Nardon et al., 2024).

While these effects are observed in healthy populations, neuromuscular fatigability can emerge more rapidly and with greater functional impact in individuals with neurological conditions (Søgaard et al., 2006; Anton et al., 2008), such as stroke (Romani, 2008; Kotwani et al., 2018), multiple sclerosis (Royer et al., 2022), or spinal cord injury (Anton et al., 2017). This stems from central and peripheral pathophysiology, including reduced voluntary drive, disrupted motor unit recruitment, and inefficient cortical activation that often requires greater neural effort to perform basic motor tasks. Peripheral changes like muscle atrophy, fiber-type shifts, and impaired neuromuscular transmission further exacerbate functional decline (Dobkin, 2008). Although patients may continue to perform functional activities like walking or standing, motor performance is frequently sustained through compensatory strategies, reflected in increased energy cost, slower pace, intermittent rest periods, and reliance on compensatory motor strategies (Dobkin, 2008). Such impairments are particularly critical in neurological rehabilitation, where efficient and adaptive motor performance is necessary for meaningful functional recovery (Juárez-Belaúnde et al., 2021).

Despite extensive work characterizing the behavioral and muscular consequences of fatigue (Abd-Elfattah et al., 2015; Patejdl and Zettl, 2022; Casamento-Moran et al., 2023), considerably less is known about how the nervous system adapts its control strategies at the level of brain–muscle communication when motor output becomes compromised. Corticomuscular coherence (CMC), the frequency-specific coupling between cortical activity (measured by electroencephalography (EEG)) and agonist muscle activation (measured by electromyography (EMG)), offers a unique, non-invasive marker of this brain–muscle interaction (Gerloff et al., 2006; Tecchio et al., 2006; Tecchio et al., 2008; Airaksinen et al., 2015). This synchrony reflects functional coupling within the descending corticospinal pathways, capturing how oscillatory synchronization between brain and muscle is modulated to support motor performance, making it particularly suited to study neuromuscular fatigue, a condition in which motor control becomes progressively challenged.

CMC has emerged as a promising biomarker of motor performance (Maura et al., 2023), displaying differences between healthy individuals and individuals with a neurological disorder (Fang et al., 2009; Pichiorri et al., 2023). In healthy individuals, CMC is typically strongest in the beta band (13–30 Hz) during steady contractions and in the gamma band (>30 Hz) during dynamic, high-intensity tasks (Liu et al., 2019). Whereas the alpha band CMC (8–12 Hz) is related to the precise control of movements and muscle readiness (Omlor et al., 2011; Stokkermans et al., 2023). Compared to healthy individuals, individuals with stroke exhibit reduced beta and gamma band CMC during movement compared to controls (Fang et al., 2009; Gao et al., 2024), and individuals with amyotrophic lateral sclerosis demonstrate diminished beta band coherence even under light loads (Proudfoot et al., 2018). Recovery of CMC strength has also been found to be linear with improvements in motor function following stroke (von Carlowitz-Ghori et al., 2014). Similarly, in Parkinson’s disease, CMC amplitude is reduced in unmedicated patients (Pollok et al., 2012). Together, these findings highlight that CMC is sensitive not only to motor impairment but also to changes in motor control strategies and recovery processes.

Importantly, neuromuscular fatigability represents a condition in which the corticospinal system is progressively stressed, potentially requiring adaptive or compensatory reorganization of neural control. As fatigue develops, maintaining motor performance may involve altered cortical synchronization, increased reliance on alternative frequency bands, or redistribution of control toward subcortical and spinal mechanisms. Characterizing these adaptations is particularly relevant for rehabilitation, where interventions aim not only to restore strength but also to promote efficient and sustainable motor control. However, existing studies (Yang et al., 2009; Tuncel et al., 2010; Yang et al., 2010; Ushiyama et al., 2011; Ushijima et al., 2017; Igasaki et al., 2018; Qiu et al., 2018; Igasaki et al., 2019; Martínez-Aguilar and Gutiérrez, 2019; Wang et al., 2020; Forman et al., 2022; Hsu et al., 2023; Correia et al., 2024; Magnuson et al., 2024; Bedard et al., 2025) report heterogeneous findings; some showing increases in coherence, others reporting decreases, and others indicating dynamic shifts depending on factors such as task characteristics, muscle group, or stage of fatigue. This variability suggests that fatigue-related changes in CMC may reflect adaptive neural strategies rather than a unidirectional loss of motor drive, underscoring the need for a systematic synthesis of literature.

The research question of this systematic review is therefore to investigate how neuromuscular fatigability influences CMC between brain-EEG activity and muscle-EMG activity in both healthy individuals and individuals with neurological disorders. By integrating evidence across tasks, contraction intensities, and populations, this review aims to clarify whether and how changes in CMC reflect compensatory neural mechanisms that support motor performance as fatigue develops during task performance. Addressing these questions will advance our understanding of the neurophysiological adaptations associated with neuromuscular fatigability in both healthy and clinical populations. These insights have the potential to inform novel strategies for rehabilitation, neuromodulation, and fatigue management in individuals with neurological disorders. A deeper understanding of these interactions will advance our knowledge of the neurophysiological adaptations associated with neuromuscular fatigability and may inform the design of future rehabilitation and neuromodulation trials, help identify neural targets sensitive to fatigue, and guide the selection of task parameters that optimally challenge or support motor control in both healthy and clinical populations.

## 2 Methods

This systematic review was registered on PROSPERO (ID: CRD42024622358) and follows the guidelines of the Preferred Reporting Items for Systematic Reviews and Meta-Analysis statement (PRISMA) (Moher et al., 2009). As no new data were collected from human participants, ethical approval was not required in accordance with institutional and international research ethics guidelines.

### 2.1 Literature search

MEDLINE (via PubMed interface), Web of Science, and Embase (via embase.com interface) were searched for relevant studies published until 02/02/2026. The same set of keywords was used across all three electronic databases to construct the search string (**Appendix 1**).

### 2.2 Selection criteria

The eligibility of the studies for inclusion in this systematic review was evaluated by two double-blinded researchers (*S.A.O. & C.M.*). All articles were screened using Rayyan (Ouzzani et al., 2016), a free web-based software tool that streamlines the process of conducting systematic reviews by facilitating the detection and elimination of duplicate and irrelevant articles and the selection of appropriate studies (Ouzzani et al., 2016). Conflicts were resolved by consensus or after consulting a third independent researcher (*M.F.*).

#### 2.2.1 Inclusion and exclusion criteria

Studies were included if they met the following criteria: participants were human adults aged 18 years or older, included either healthy individuals or individuals with a neurological disorder, assessed neuromuscular fatigability using both peripheral fatigability metrics derived from EMG and central fatigability metrics derived from EEG, and reported changes in CMC measures in response to fatigue. In this review, neuromuscular fatigability refers specifically to the exercise- or task-induced reduction in the muscle’s ability to generate force, due to changes within the central and/or peripheral nervous system (Enoka and Duchateau, 2016). This excludes other forms of fatigue, such as psychological or cognitive fatigue. Only peer-reviewed, full-text original experimental studies published in English were considered for inclusion, including within-subject, repeated-measures, or crossover designs that investigated CMC during fatiguing tasks. Studies were excluded if they involved athletes defined as individuals engaged in regular, structured, high-frequency training for competitive purposes or performance-based recognition, as such training is known to alter motor unit behaviour and neuromuscular and corticospinal adaptations (Moscatelli et al., 2016; Maeo et al., 2021; Škarabot et al., 2024). Additional exclusions applied to studies: focused only on other forms of state fatigue (e.g., cognitive or mental fatigue) or trait fatigue (e.g., post-stroke fatigue or multiple sclerosis-related fatigue); measured central fatigability using solely other methods (e.g., near-infrared spectroscopy, functional magnetic resonance imaging) or peripheral fatigability through measures such as force output or dynamometry; reporting directional or nonlinear coupling metrics (e.g., transfer entropy, partial directed coherence, mutual information) without coherence-based CMC measures; involved participants with comorbidities as musculoskeletal injuries, or severe metabolic or cardiopulmonary conditions; employed combined modalities or interventions, such as resistance exercises, electrical stimulation, or pharmacological interventions.

### 2.3 Quality assessment

The risk of bias in included studies was assessed independently by two reviewers (*S.A.O. and A.D.*) using the ROBINS-I tool (Risk of Bias in Non-randomized Studies of Interventions) version 2. This tool evaluates the risk of bias due to confounding, selection of participants into the study, classification of interventions, deviations from intended interventions, missing data, measurement of outcomes, and selection of the reported results (Sterne et al., 2016). Each domain was rated as low, moderate, serious, or critical risk of bias. Disagreements between reviewers were resolved through discussion or consultation with a third reviewer (*M.F.*).

### 2.4 Data extraction

The following data were extracted from each article: author, year of publication, study design, subject characteristics, fatiguing task characteristics, outcome measures, and key findings regarding the changes of CMC measures related to neuromuscular fatigability, including *p*-values.

## 3 Results

**Figure 1:**
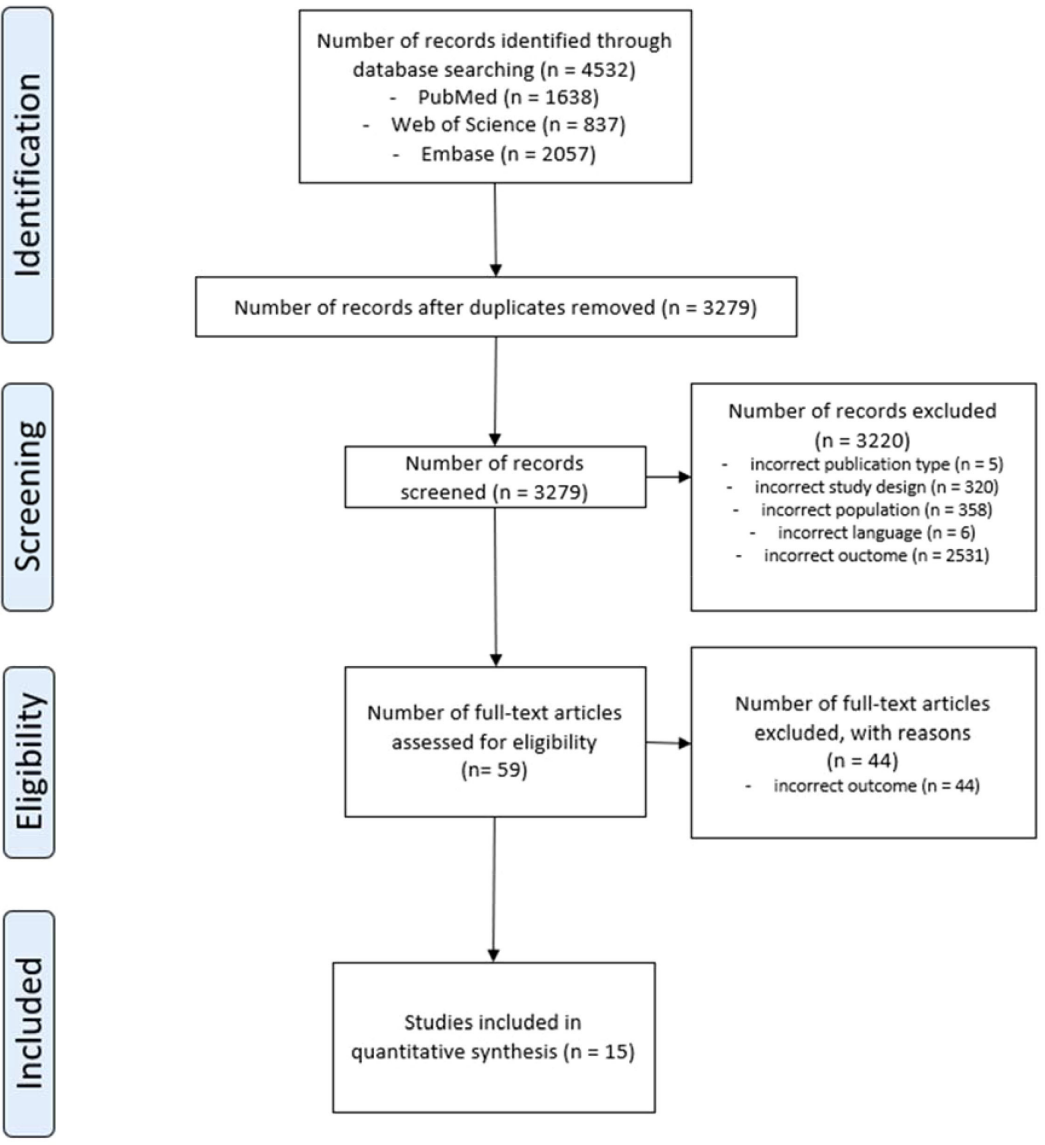
PRISMA flow diagram depicting the study selection process.

A total of 3,279 studies were screened after deduplication; 59 studies were selected based on title and abstract, and 15 were finally included after full-text screening. Details can be found in the flowchart (**Figure 1**).

### 3.1 Characteristics of included studies

A total of 15 non-randomized trials examining the relationship between neuromuscular fatigability and CMC were included. Only one of the included studies involved a neurological population (adults with cerebral palsy), while the remaining were healthy. Across studies, 188 participants were enrolled: 174 neurologically healthy adults (137 males, 37 females) and 14 individuals with neurological conditions (7 males, 7 females). Overall, participants were predominantly healthy young to middle-aged males, with reported mean ages ranging from 22.7 to 48.2 years (**Table 1**). Considerable heterogeneity existed across studies in terms of fatiguing task characteristics, fatigue assessment timepoints, and CMC analysis methods (**Tables 1**, **2**). Existing studies examining fatigue-related alterations in brain–muscle communication in neurological populations were largely not included in the present review because they did not meet the predefined inclusion criteria of investigating experimentally-induced neuromuscular fatigue, focusing solely on neuromuscular fatigability (state fatigue), or tracking the modulation of CMC with respect to fatigability (e.g., pre–post task assessment).

**Table 1:** Summary of findings.

Study	Participants	Fatiguing task			CMC	Timepoints	Key findings
	Sex				Cortical area		
	Age ± SD	Intensity	Type	Mode	Muscle		
(Bedard et al., 2025)	14 ♂, 5 ♀	Handgrip 50% MVC	Isometric	Intermittent	C3	Across (pre-NMF& post-NMF) & within 8 blocks	mean β & γ CMC no sign. diff. post- vs pre-NMF (*p* > 0.05) β & γ CMC sign. ↑ within blocks *(p* < 0.05)
	44 ± 13 y				R FCR_m_ & ECR_m_		
(Correia et al., 2024)	19 ♂	Knee extension & flexion	Isotonic	Intermittent	C3	Early, mid, late NMF	early vs. late NMF: Coharea sign. ↓ across all muscles (*p* < 0.05)
	25.9 ± 6.4 y				R VL_m_, RF_m,_ VM_m_, BF_m_, & ST_m_		
(Magnuson et al., 2024)	8 ♂, 8 ♀	Elbow Flexion 20% MVC	Isometric	Sustained	Cz	Pre-NMF, Post-NMF	mean α CMC sign. ↑ post NMF mean β CMC no sign. diff. post- vs pre-NMF (*p* > 0.05)
	28.0 ± 4.3 y				BB_m_		
(Hsu et al., 2023)	7♂, 6♀	Handgrip 3x 60% MVC + 1x 100% MVC	Isometric	Intermittent	C3	Pre-NMF, Post-NMF	Coharea: β CMC sign. ↑ post- vs pre-NMF (*p* < 0.05) Coharea: γ CMC: no sign. diff. post- vs pre-NMF
	25.3 ± 2.5 y				R FDS_m_		
(Forman et al., 2022)	Cerebral Palsy: 7♂, 7♀ 37.6 ± 10.1 y	Dorsiflexion 30- 60% MVC	Isometric	Sustained	FC/C/CP	Pre-NMF, Post-NMF	CP & NI : AUC β CMC: sign. ↓ post-NMF (*p* < 0.05): CP < NI (*p* < 0.05) AUC α & γ CMC: no sign. diff. (all *p’s > *0.05)
	Control: 4♂, 6♀ 35.4 ± 10.3 y				R & L TA_m_		
(Igasaki et al., 2019)	7♂	Dorsiflexion 10–60% MVC	Isometric	Sustained	Cz	5 epochs (every 12’’, incl. pre- and post-NMF)	10–30% MVC: PCF: sustained β CMC but ↓ PCV over time ≥40% MVC: early PCF: γ CMC but later PCF: β CMC
	23 ± 1 y				R GC_m_		
(Wang et al., 2020)	12♂	Elbow Extension 20% MVC	Isometric	Sustained	C3	Pre-NMF, Post-NMF	Coharea CMC observed in α, β & γ γ CMC sign. ↓ post- vs pre-NMF (*p* < 0.05)
	23.75 ± 2.49 y				R TB_m_		
(Martínez-Aguilar and Gutiérrez, 2019)	5♂, 5♀	Pedaling 5’ TF	Isotonic	Intermittent	F3, Fz, F4, C3, Cz, C4, P3, Poz, P4	Pre-NMF, Post-NMF	PCV α CMC: no sign. diff. post- vs pre-NMF PCV β CMC: sign. diff. post- vs pre-NMF across subjects
	26.8 ± 3.4 y				L VL_m_		
(Igasaki et al., 2018)	5♂	Dorsiflexion 10–60% MVC	Isometric	Sustained	Cz	5 epochs (every 12’’, incl. pre- and post-NMF)	10–30% MVC: PCF: sustained β CMC, ↓ PCV over time 40–60% MVC: no sign. PCF CMC (sign γ CMC: first 12’’ only) no sign. diff. in PCV or PCF across time divisions in any frequency band (all *p’s >* 0.05)
	23 ± 1 y				R TA_m_		
(Qiu et al., 2018)	18♂	Shoulder Abduction 0–3kg	Isometric	Sustained	C3, C4, CP5, CP6	Pre-NMF, Post-NMF	γ Coharea sign. ↓ post-NMF (all *p*’s < 0.05) no sign. effect of force load on CMC in any frequency band (*p* > 0.05)
	25 ± 3 y				R D_m_		
(Ushijima et al., 2017)	11♂	Dorsiflexion 10–30% MVC	Isometric	Sustained	19 scalp electrodes	5 epochs (every 12’’, incl. pre- and post-NMF)	β PCV sign. ↑ with post-NMF (*p* < 0.05) γ CMC no sign. diff. post-NMF (*p* > 0.05) PCF: remained mostly in β (*p* > 0.05)
	25 ± 4 y				R TA_m_		
(Ushiyama et al., 2011)	7♂	Dorsiflexion 30–50% MVC	Isometric	Sustained	Cz + 20mm L	Pre-NMF, Post-NMF	β CMC sign. ↑ post-NMF (Cohmax *p* < 0.05; Coharea: *p* < 0.05)
	22.7 ± 1.6 y				R TA_m_		
(Tuncel et al., 2010)	10♂	Elbow Flexion 4 kg	Isometric	Intermittent	C3-C4	Early, mid, late NMF	At early and mid-NMF: sign. β & γ CMC mean values At late-NMF: γ CMC & ↓ β & γ CMC mean values
	± 23 y				R BB_m_		
(Yang et al., 2010)	7♂, 1♀	Handgrip 200 x 100% MVC	Isometric	Intermittent	C3, C4, Cz, Fz, Pz	150 trials (incl. pre-NMF and post-NMF)	mean β & γ CMC sign. ↓ post- vs pre-NMF
	32.7 ± 7.7 y				R FDP_m_ & FDS_m_		
(Yang et al., 2009)	3♂, 6♀	Elbow Flexion 30% MVC	Isometric	Sustained	F3, Fz, F4, C3, Cz, C4, P3, Pz, P4	Minimal & Severe NMF	PCV β CMC sign. ↓ with severe NMF (*p* < 0.05) PCF remained in β with NMF
	48.2 ± 14.8 y				R BB_m_		

y, years; MVC, Maximum Voluntary Contraction, L, Left; R, Right; C3, Left Motor Area; C4, Right Motor Area; Cz, Central Region; F3, Left Frontal Cortex; Fz, Midline Frontal Cortex; F4, Right Frontal Cortex; P3, Left Parietal Cortex; Poz, Midline Parietal Cortex; P4, Right Parietal Cortex; CP5, Left Centro-Parietal Regions; CP6, Right Centro-Parietal Regions; FCR_m_, Flexor Carpi Radialis; ECR_m_, Extensor Carpi Radialis muscle; VL_m_, Vastus Lateralis muscle; RFm, Rectus Femoris muscle; VM_m_, Vastus Medialis muscle; BF_m_, Biceps Femoris muscle; ST_m_, Semitendinosus muscle; BB_m_, Biceps Brachii muscle; FDS_m_, Flexor Digitorum Superficialis muscle; TA_m_, Tibialis Anterior muscle; GC_m_, Gastrocnemius muscle; TB_m_, Triceps brachii muscle; D_m_, Deltoid muscle; FDP_m_, Flexor Digitorum Profundus muscle; NMF, Neuromuscular Fatigue; PCV, Peak Coherence Value; PCF, Peak Coherence Frequency; Cohmax, Coherence Maximum; Coharea, Coherence Area; AUC, Area Under the Curve; diff, difference; α, Alpha Band; β, Beta Band; γ, Gamma Band; sign., Significant; ↑, Increase; ↓, Decrease; CMC, Corticomuscular Coherence, *p*, Statistical Significance.

**Table 2:** Different corticomuscular coherence outcomes reported across the studies.

Outcome	Definition
Peak Coherence Value (*PCV*) / Coherence Maximum (*Cohmax)*	The maximum level of synchronization or correlation observed between two signals within a specified frequency range (Yang et al., 2009; Ushiyama et al., 2011; Ushijima et al., 2017; Igasaki et al., 2018; Igasaki et al., 2019; Martínez-Aguilar and Gutiérrez, 2019)
Peak Coherence Frequency (*PCF*)	The exact frequency within a band where CMC reaches its maximum value (PCV) between EEG and EMG (Yang et al., 2009; Igasaki et al., 2018; Igasaki et al., 2019; Martínez-Aguilar and Gutiérrez, 2019)
Coherence Area (*Coharea*) / Area under the curve	The total coherence between two signals across a specified frequency range, calculated as the integral of the coherence spectrum (Tuncel et al., 2010; Ushiyama et al., 2011; Qiu et al., 2018; Wang et al., 2020; Forman et al., 2022; Hsu et al., 2023; Correia et al., 2024)
Mean CMC	Average coherence across all frequency bins within a specified frequency range (Yang et al., 2010; Bedard et al., 2025)
Wavelet Coherence	Quantifies the linear correlation between two time series (e.g., EEG and EMG signals) in the time-frequency domain (Yang et al., 2010)

### 3.2 Quality analysis

**Figure 2:**
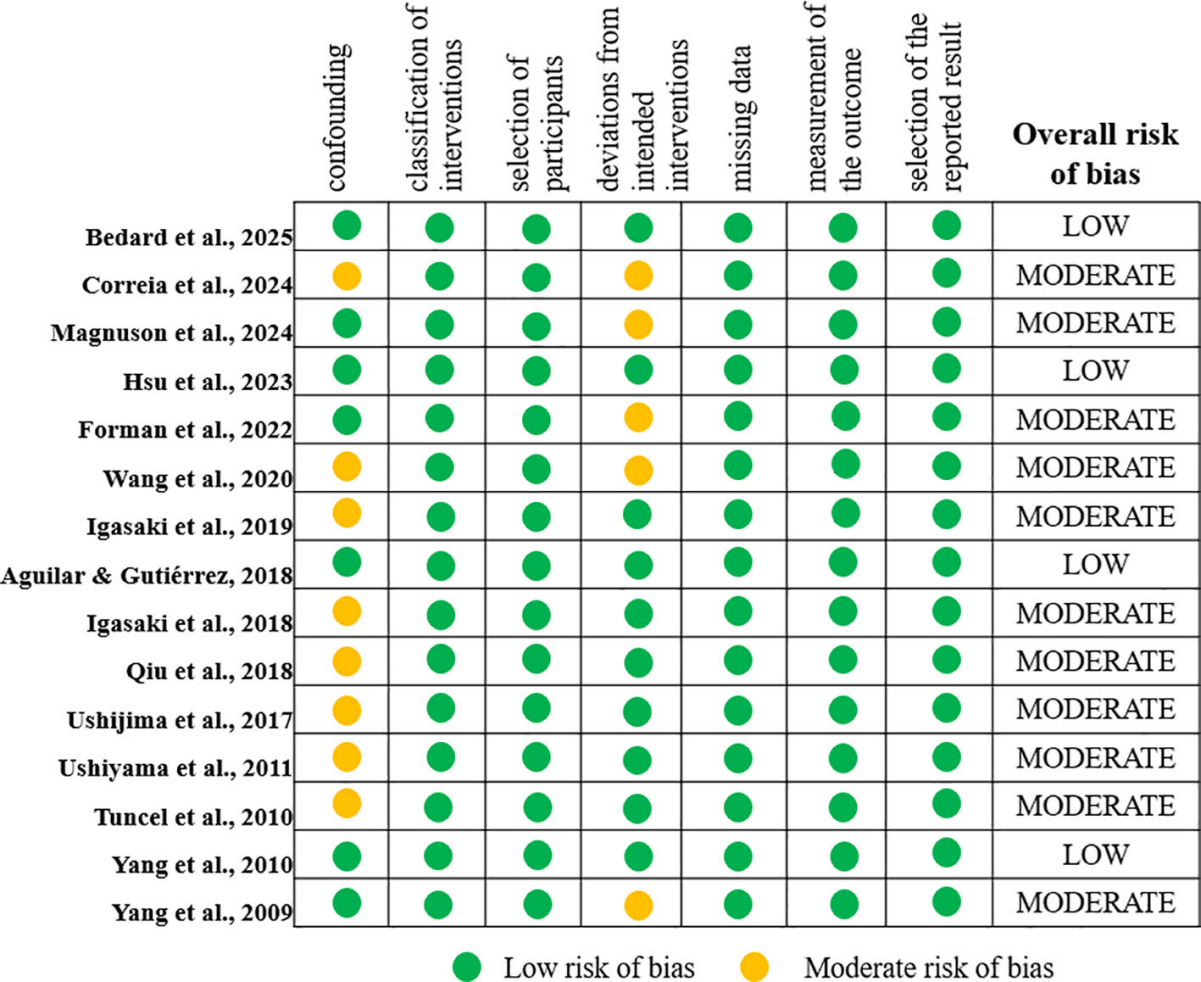
Risk of bias and overall quality for individual studies.

Out of the included studies, four were rated as having a low risk of bias across all ROBINS-I domains (Yang et al., 2010; Martínez-Aguilar and Gutiérrez, 2019; Hsu et al., 2023; Bedard et al., 2025). These studies demonstrated minimal concern for confounding or missing data. The remaining studies (Yang et al., 2009; Tuncel et al., 2010; Ushiyama et al., 2011; Ushijima et al., 2017; Igasaki et al., 2018; Qiu et al., 2018; Igasaki et al., 2019; Wang et al., 2020; Forman et al., 2022; Correia et al., 2024; Magnuson et al., 2024) were rated as having a moderate risk of bias. Common methodological limitations included limited generalizability due to male-only samples or potential biases in outcome measurement (**Supplementary Material**, **Figure 2**). Nevertheless, these studies retained sufficient methodological integrity to contribute meaningfully to the synthesis. No studies were rated as having a serious or critical risk of bias.

### 3.3 CMC analysis

Across the included studies, alpha band CMC was most commonly defined as 8–12 Hz (Martínez-Aguilar and Gutiérrez, 2019; Wang et al., 2020; Hsu et al., 2023; Magnuson et al., 2024), with broader definitions also reported, including 6–15 Hz (Forman et al., 2022) and 8–13 Hz (Bedard et al., 2025); the beta band generally spanned 13–35 Hz, most frequently 15–35 Hz (Yang et al., 2009; Yang et al., 2010; Igasaki et al., 2018; Igasaki et al., 2019; Wang et al., 2020; Magnuson et al., 2024), with narrower or shifted ranges such as 13–29 Hz (Martínez-Aguilar and Gutiérrez, 2019), 13–30 Hz (Ushiyama et al., 2011; Ushijima et al., 2017; Qiu et al., 2018; Hsu et al., 2023; Bedard et al., 2025), 16–35 Hz (Forman et al., 2022), and 15–30 Hz (Tuncel et al., 2010); and the gamma band covered approximately 30–60 Hz, commonly 35–60 Hz (Igasaki et al., 2018; Igasaki et al., 2019; Wang et al., 2020), with alternative definitions including 36–60 Hz (Forman et al., 2022), 30–60 Hz (Tuncel et al., 2010; Ushijima et al., 2017), 30–50 Hz (Hsu et al., 2023; Bedard et al., 2025), 30–45 Hz (Qiu et al., 2018), and 35–50 Hz (Yang et al., 2010).

CMC was predominantly calculated between EEG electrodes over the sensorimotor cortex and surface EMG signals recorded from task-relevant muscles of the active limb. Upper-limb studies typically assessed CMC between contralateral central electrodes (mostly C3) and elbow, forearm, or hand muscles. Lower-limb studies primarily computed CMC between Cz or neighboring central electrodes and knee or ankle muscles. Some studies extended the analysis to multiple scalp regions (frontal, central, and parietal) to capture distributed corticomuscular interactions (Yang et al., 2009; Yang et al., 2010; Ushijima et al., 2017; Qiu et al., 2018; Martínez-Aguilar and Gutiérrez, 2019; Forman et al., 2022) (**Table 2**).

#### 3.3.1 Frequency-domain CMC analysis

Across 11 included studies (Yang et al., 2009; Ushiyama et al., 2011; Ushijima et al., 2017; Igasaki et al., 2018; Qiu et al., 2018; Igasaki et al., 2019; Martínez-Aguilar and Gutiérrez, 2019; Wang et al., 2020; Forman et al., 2022; Hsu et al., 2023; Magnuson et al., 2024), CMC was computed using Fourier-based magnitude-squared coherence derived from EEG–EMG cross-spectral densities averaged over time windows. These studies typically assume signal stationarity within the analyzed segment and report outcomes such as mean CMC values (Yang et al., 2009; Igasaki et al., 2018; Qiu et al., 2018; Wang et al., 2020; Magnuson et al., 2024), peak coherence value (PCV)/coherence maximum (Cohmax) (Ushiyama et al., 2011; Ushijima et al., 2017; Igasaki et al., 2018; Igasaki et al., 2019; Martínez-Aguilar and Gutiérrez, 2019), peak coherence frequency (PCF) (Yang et al., 2009; Ushiyama et al., 2011; Ushijima et al., 2017; Igasaki et al., 2018; Igasaki et al., 2019; Martínez-Aguilar and Gutiérrez, 2019; Wang et al., 2020), or the area of coherence (Coharea) (Ushiyama et al., 2011; Qiu et al., 2018; Wang et al., 2020; Forman et al., 2022; Hsu et al., 2023) exceeding a statistical threshold (95% confidence limit) (**Table 2**). CMC values range between 0 and 1, with 0 indicating no linear relationship between EEG and EMG at that frequency and 1 indicating a perfect linear relationship. The 95% confidence limit is applied to this 0–1 range to identify which coherence values are statistically significant. Under this framework, fatigue-related changes are interpreted as net pre-to-post or early-to-late differences. Across these studies, CMC between an EEG signal x(t) and an EMG signal y(t) was computed as:


$${\text{CMC}}_{xy}(f)=\frac{|S_{xy}(f){|}^{2}}{S_{xx}(f)\;S_{yy}(f)}$$

where $S_{xy}(f)$ represents the cross-spectral density between EEG and EMG at frequency *f*, and $S_{xx}(f)$ and $S_{yy}(f)$ represent their respective auto-spectral densities.

#### 3.3.2 Time–frequency CMC analysis

Four studies extended the Fourier-based framework into the time–frequency domain (Tuncel et al., 2010; Yang et al., 2010; Correia et al., 2024; Bedard et al., 2025), allowing coherence to vary dynamically over the course of fatiguing tasks. These studies reported outcomes such as band-mean coherence (Bedard et al., 2025), the summed or averaged area of significant coherence above a statistical threshold (Tuncel et al., 2010; Correia et al., 2024), or wavelet coherence (Yang et al., 2010) within predefined frequency bands, capturing transient changes that occur during non-stationary muscle activity (**Table 2**). Similar to frequency-domain CMC, coherence values ranged from 0 to 1, with 0 indicating no linear relationship and 1 indicating perfect linear coupling between EEG and EMG at a given time–frequency point. Statistical significance was determined using a 95% confidence limit.

### 3.4 Changes in CMC related to neuromuscular fatigability

#### 3.4.1 Changes in alpha band CMC (~8–12 Hz)

Alpha band CMC was only reported in four studies, with inconsistent modulation from pre- to post-fatigue states or across task stages. Specifically, no fatigue-related differences in alpha CMC were observed during cyclic knee movements (Martínez-Aguilar and Gutiérrez, 2019) or during sustained ankle dorsiflexion in both neurologically intact individuals and individuals with cerebral palsy (Forman et al., 2022) (all *p’s > 0.05)*. Similarly, Wang et al (Wang et al., 2020) reported the presence of alpha band CMC during elbow extension tasks, but no significant alpha band modulation with fatigability, despite observing significant changes in other frequency bands. In contrast, Magnuson et al (Magnuson et al., 2024) reported a significant increase in alpha band CMC with neuromuscular fatigability during a sustained contraction (*p < 0.05*). Overall, alpha band CMC was either unchanged or only partially affected by fatigability, with significant effects reported in isolated task conditions rather than consistently across studies.

#### 3.4.2 Changes in beta band CMC (~13–35 Hz)

Beta band CMC showed non-uniform modulation with neuromuscular fatigability. Apparent inconsistencies largely reflect differences in fatiguing tasks, fatigue assessment timepoints, CMC analysis method, and population studied. Recent work by Bedard et al (Bedard et al., 2025) demonstrated that beta band CMC increased transiently within individual task blocks (repetitions), with positive and significant slopes in nearly all blocks (*p ≤ 0.05*). However, this increase did not persist across blocks, as the average beta CMC amplitude did not change over the course of the task (*p > 0.05*), showing short-term adjustments rather than cumulative increases. This within-block modulation aligns with earlier studies reporting increases in beta band CMC during or immediately after fatiguing contractions, such as those by Hsu et al (Hsu et al., 2023), Ushijima et al (Ushijima et al., 2017), and Ushiyama et al (Ushiyama et al., 2011), who observed higher beta band CMC in post-fatigue or late-fatigue states (*p < 0.05*).

In contrast, studies focusing on later phases of prolonged or repetitive tasks consistently report reductions in beta CMC. Correia et al (Correia et al., 2024) showed a significant decrease in the area of significant CMC during the second half of the task across multiple muscles (all *p ≤ 0.05*), a pattern also observed by Wang et al (Wang et al., 2020), Aguilar & Gutiérrez (Martínez-Aguilar and Gutiérrez, 2019), Tuncel et al (Tuncel et al., 2010), and Yang et al (Yang et al., 2009; Yang et al., 2010), where beta band CMC was stronger early and declined as fatigue developed (*p < 0.05).* Consistent with this pattern, Igasaki et al. demonstrated that beta band CMC was dominant during weak contractions (10–30% MVC) (Igasaki et al., 2019) but declined over time (Igasaki et al., 2018), whereas during stronger contractions (40–60% MVC), beta band CMC was less significant. Importantly, Magnuson et al (Magnuson et al., 2024) reported no significant pre-to-post changes in beta band CMC during a sustained 10-min contraction (*p > 0.05*). Evidence from a single neurological population (Forman et al., 2022) reported a significant reduction in beta band CMC with neuromuscular fatigability (*p < 0.05*), with individuals with cerebral palsy showing lower beta CMC than neurologically intact controls (*p < 0.05*).

#### 3.4.3 Changes in gamma band CMC (~30–60 Hz)

Gamma band CMC appears less variable than beta band CMC and shows relatively more consistent modulation with fatigability. Recent evidence from Bédard et al (Bedard et al., 2025). indicates that gamma band CMC can increase transiently within task blocks, mirroring the same within-block behavior observed in the beta band. However, these transient increases did not translate into significant changes across blocks, as block-averaged gamma band CMC amplitude remained stable despite the progressive development of fatigue. This finding aligns with several earlier studies reporting no significant pre-to-post or time-dependent changes in gamma band CMC (Ushijima et al., 2017; Hsu et al., 2023), including Forman et al (Forman et al., 2022), who observed no significant differences in the cerebral palsy group in association with neuromuscular fatigability (all *p’s > 0.05*).

Compared with this, multiple studies documented significant reductions or disappearance of gamma band CMC as fatigue progressed (Tuncel et al., 2010; Yang et al., 2010; Igasaki et al., 2018; Qiu et al., 2018; Igasaki et al., 2019; Wang et al., 2020), particularly during prolonged or stronger contractions (Tuncel et al., 2010; Igasaki et al., 2018; Igasaki et al., 2019), with gamma coherence often appearing only briefly at task onset and declining as fatigue increased.

#### 3.4.4 Limb-related CMC changes with neuromuscular fatigability

The studies included were further grouped by limb and muscle group. Different fatigue-related CMC changes were observed across the upper- (Yang et al., 2009; Tuncel et al., 2010; Yang et al., 2010; Qiu et al., 2018; Wang et al., 2020; Hsu et al., 2023; Magnuson et al., 2024; Bedard et al., 2025) and lower- (Ushiyama et al., 2011; Ushijima et al., 2017; Igasaki et al., 2018; Igasaki et al., 2019; Martínez-Aguilar and Gutiérrez, 2019; Forman et al., 2022; Correia et al., 2024) limb tasks.

Upper-limb fatiguing tasks (8 studies) showed heterogeneous CMC responses across shoulder, elbow, and hand/forearm muscles. During shoulder abduction involving the deltoid, gamma band CMC decreased significantly with neuromuscular fatigability, with no effect of force load on coherence in any frequency band (Qiu et al., 2018). For the biceps brachii muscle, mixed results were reported: alpha band CMC increased post-fatigue, while beta band CMC showed either no significant pre-to-post fatigue change (Magnuson et al., 2024) or significant reductions with greater neuromuscular fatigability (Yang et al., 2009) along with gamma band CMC (Tuncel et al., 2010). Whereas only the gamma band CMC decreased for the triceps brachii muscle (Wang et al., 2020). For hand and forearm muscles, block-designed handgrip task showed no significant beta or gamma band CMC differences pre-to-post fatigue (Bedard et al., 2025), whereas sustained handgrip demonstrated either significant increases in beta band CMC (Hsu et al., 2023) or significant reductions in both beta and gamma band CMC across flexor digitorum superficialis and profundus (Yang et al., 2010). For lower-limb muscles (7 studies), fatigue-related CMC changes were more consistently characterized by reductions in coherence across fatigue assessment timepoints and contraction intensity. During knee flexion-extension tasks involving the vastus lateralis, rectus femoris, vastus medialis, biceps femoris, and semitendinosus muscles, a significant decrease in CMC was observed from early to late fatigue across all recorded muscles (all *p’s <0.05*) (Correia et al., 2024). In a pedaling fatiguing task assessing the vastus lateralis muscle, alpha band CMC showed no significant pre-to-post fatigue change, whereas beta band CMC differed significantly between subjects with different levels of fatigability (Martínez-Aguilar and Gutiérrez, 2019). For the tibialis anterior muscle, results varied with contraction intensity: during low-intensity contractions (10–30% MVC), significant beta band CMC was observed but declined over time, while high-intensity contractions (40–60% MVC) showed no sustained CMC, with gamma band coherence present only transiently during the initial seconds (Igasaki et al., 2018). Only two studies reported significant post-fatigue increases in beta band CMC irrespective of contraction intensity, with coherence frequency remaining in the beta range (Ushiyama et al., 2011; Ushijima et al., 2017). In the clinical population, beta band CMC decreased significantly after a fatiguing dorsiflexion task for the tibialis anterior muscle, with lower coherence values in individuals with cerebral palsy compared with neurologically intact controls (Forman et al., 2022). For the gastrocnemius muscle, weak contractions (10–30% MVC) showed sustained beta band CMC, whereas stronger contractions (≥40% MVC) were initially dominated by gamma band CMC that declined over time (Igasaki et al., 2019).

#### 3.4.5 Task-related CMC changes: contraction type, mode, and intensity

CMC responses to neuromuscular fatigability varied according to task characteristics, particularly contraction type, mode, and intensity.

Studies using fatiguing intermittent or block-designed isometric tasks reported mixed CMC responses. Bédard et al (Bedard et al., 2025) found no significant pre-to-post fatigue changes in block-averaged beta or gamma CMC (*p > 0.05*), whereas Hsu et al (Hsu et al., 2023). reported a significant increase in beta band CMC post-fatigue (*p < 0.05*) with no change in gamma band CMC. Yang et al (Yang et al., 2010) reported significant post-fatigue reductions in both beta and gamma band CMC compared with pre-fatigue values. Similarly, Tuncel et al (Tuncel et al., 2010) observed significant beta and gamma band CMC during early and mid-fatigue stages, followed by a reduction in both bands during late fatigue, with gamma band coherence remaining significant. Two studies using cyclic isotonic fatiguing contractions reported no changes in CMC. Aguilar & Gutiérrez (Martínez-Aguilar and Gutiérrez, 2019) reported no significant pre-to-post fatigue changes in alpha band CMC and significant differences in beta band CMC, but the direction (increase vs decrease) varied between individuals. In contrast, Correia et al (Correia et al., 2024) demonstrated a significant decrease in CMC from early to late fatigue stages across all recorded muscles (all *p’s < 0.05*).

In studies using isometric tasks, including handgrip (Yang et al., 2010; Hsu et al., 2023; Bedard et al., 2025), elbow flexion/extension (Yang et al., 2009; Tuncel et al., 2010; Wang et al., 2020; Magnuson et al., 2024), and ankle dorsiflexion (Ushiyama et al., 2011; Ushijima et al., 2017; Igasaki et al., 2018; Igasaki et al., 2019; Forman et al., 2022), significant fatigue-related changes in beta band CMC were frequently reported. During low-intensity sustained contractions (10–30% MVC), results were mixed. Ushijima et al (Ushijima et al., 2017) reported significant increases in beta band CMC with fatigability, whereas other studies at similar force levels observed significant reductions in beta band CMC (Yang et al., 2009; Igasaki et al., 2019) and gamma band CMC (Qiu et al., 2018; Wang et al., 2020) during later task stages. Magnuson et al (Magnuson et al., 2024) found no significant changes in beta band CMC, with only alpha band CMC showing an increase. During higher intensity or prolonged contractions to task failure (40–60% MVC or repeated maximal efforts), beta band CMC showed generally attenuated responses with fatigability. Ushiyama et al (Ushiyama et al., 2011) reported a significant increase in beta band CMC post-fatigue at 30–50% MVC (Cohmax and Coharea, both *p’s < 0.05*), with beta frequencies present both pre- and post-fatigue. Conversely, Forman et al (Forman et al., 2022) observed a significant post-fatigue reduction in beta band CMC (*p < 0.05*), with lower values in individuals with cerebral palsy compared with neurologically intact controls (*p < 0.05*), while alpha- and gamma band CMC showed no significant changes. Igasaki et al (Igasaki et al., 2019) reported that strong contractions (≥40% MVC) were characterized by an initial gamma band CMC that decreased over time, whereas in their earlier work (Igasaki et al., 2018) no significant sustained CMC at high intensities (40–60% MVC) was found, with gamma band coherence present only transiently during the first 12 s.

## 4 Discussion

This systematic review included 15 non-randomized studies investigating how neuromuscular fatigability affects brain–muscle synchronization. The primary outcome was changes in CMC associated with neuromuscular fatigability. According to the reviewed studies, alpha, beta (most prominent), and gamma bands CMC demonstrated divergent patterns as fatigue developed, influenced by several key factors. Variations in frequency band definitions did not seem to systematically affect outcomes, as similar trends were reported despite differences in band boundaries, a common methodological challenge in EEG research (Newson and Thiagarajan, 2019). In contrast, the choice of analysis approach (frequency-domain vs. time–frequency domain) may have contributed to some variability, particularly in handgrip paradigms, where different findings were noted in the beta and gamma bands, similar to alpha band results (all derived from frequency-domain analyses). A pronounced source of heterogeneity concerned the definition of fatigue phases. Studies employed a wide range of approaches, including within-task temporal segmentation, pre–post comparisons, and multi-stage fatigue classifications, often without standardized terminology or clearly defined criteria, limiting direct temporal comparisons and precluding subgroup analysis. Although research in this area is expanding, the heterogeneity of findings among the methodologies limits generalizability, and studies involving neurological populations remain notably scarce, with only one out of the 15 included studies examining a neurological population (adults with cerebral palsy).

### 4.1 Reconsidering cortical involvement: Does increased cortical activity support sustained motor performance with neuromuscular fatigability?

Beta band CMC is widely interpreted as a marker of effective corticospinal communication during steady force output and precision control (Engel and Fries, 2010; Reyes et al., 2017), and its fatigue-related modulation appears to depend on when and how fatigue was induced, as well as task characteristics and the EEG-EMG coupling analysis method employed (Liu et al., 2019) Because beta band CMC was the most examined frequency band and yielded the most interpretable fatigue-related effects, the following section exhaustively focuses on beta band CMC findings, which can be firsthand grouped by task type (dorsiflexion, handgrip, elbow flexion). In dorsiflexion studies, contraction intensity emerged as a key differentiating factor. Low- to moderate-intensity sustained contractions (≈10–30% MVC) as in Ushijima et al (Ushijima et al., 2017) and Ushiyama et al (Ushiyama et al., 2011) were associated with significant increases in beta CMC post-fatigue, which may reflect increased cortical engagement to stabilize force as peripheral fatigability emerges. This aligns with evidence that beta coherence can increase during mild fatigue, when the motor system prioritizes precision and steadiness (McManus et al., 2016). By contrast, Igasaki et al (Igasaki et al., 2018; Igasaki et al., 2019), who examined a higher intensity range up to 60% MVC using several fatigue epochs, found that beta band CMC was either reduced or diminished over time at high-intensity force levels. These findings suggest that increasing force demands as fatigue develops may overwhelm corticomuscular synchrony within beta coupling, leading to a reduced ability of the motor cortex to synchronize its output with muscle activity, a pattern consistent with reports of weakened beta coherence under high metabolic and central constraints (Tecchio et al., 2006; Pethick and Tallent, 2022). The findings by Forman et al (Forman et al., 2022) provide further support for this interpretation. The observation that neurologically intact controls also showed a reduction in beta CMC after fatigue may indicate that such a decrease represents a broader response to severe fatigue. The larger reduction reported in cerebral palsy may therefore reflect an added vulnerability associated with pre-existing alterations in corticomuscular connectivity (Meng et al., 2009; Salomon, 2024). Similar divergent patterns were observed in handgrip and elbow flexion studies, where the fatigue assessment timepoints and CMC analysis approach emerged as additional critical determinants of reported beta band CMC changes. In handgrip paradigms, Hsu et al (Hsu et al., 2023) reported increased beta band CMC when assessing only pre-to-post fatigue changes using frequency-domain analysis, whereas Bedard et al (Bedard et al., 2025) observed no net pre-to-post change despite transient within-block increases captured with time-frequency methods. However, Yang et al (Yang et al., 2010), using also time-frequency analysis with broader montage (C3, C4, Cz, Fz, Pz), reported decreases in beta band CMC, underscoring that the analytic approach (frequency vs time–frequency) may influence the detection of fatigability dynamics (Liu et al., 2019). Moreover, although differences in EEG montages (Cz vs multi-electrode arrays) may modulate the sensitivity to beta band CMC changes, this factor is more likely to influence the magnitude rather than the direction of the observed effects (Mima and Hallett, 1999) and is unlikely to account for the direction of fatigue-related changes reported in this review. Similarly, within the elbow flexion studies (Yang et al., 2009; Tuncel et al., 2010; Wang et al., 2020; Magnuson et al., 2024), reductions in beta band CMC were consistently observed despite differences in contraction mode (sustained vs intermittent), suggesting that contraction continuity alone does not fully determine the direction of beta CMC modulation as fatigue develops.

Gamma band CMC was less reported than beta band CMC across the included studies and showed predominantly no significant change or progressive reductions as fatigue develops across tasks. In sustained dorsiflexion paradigms, gamma band CMC was largely absent or unchanged across fatigue stages, although transient increases in gamma coupling have been reported during early phases of high-intensity or dynamic contractions; these effects appear short-lived and are not consistently captured in pre–post fatigue comparisons (Igasaki et al., 2018; Igasaki et al., 2019). However, in intermittent handgrip tasks, gamma band CMC decreased as contraction intensity increased (Yang et al., 2010), while it remained unchanged at lower intensity levels (Hsu et al., 2023; Bedard et al., 2025). This finding appears counterintuitive, as gamma-band CMC is typically linked to dynamic, high-force motor control and rapid sensorimotor integration (Gwin and Ferris, 2012). Importantly, gamma band CMC is inherently more challenging to detect reliably due to the lower signal-to-noise ratio (He, 2014), owing to the relatively spatially restricted generators of gamma oscillations, and their tendency to occur in brief bursts rather than sustained rhythms. Consequently, the detection of gamma activity and its associated connectivity structure is technically more demanding (Buzsáki and Wang, 2012), potentially contributing to reduced sensitivity and a higher likelihood of null findings. In addition, the type of motor task influences the frequency band in which CMC is most prominent: isotonic contractions tend to shift corticospinal oscillatory coupling toward the gamma range, whereas isometric contractions more consistently favour beta band coherence (Gwin and Ferris, 2012). Given that most included studies employed isometric contractions, this methodological characteristic may have further limited the detection of gamma band CMC. Therefore, the limited findings in the gamma band in this review are likely reflect methodological and task-related constraints rather than an absence of physiological involvement.

Crucially, weakening in beta or gamma band CMC during neuromuscular fatigability should not be interpreted as solely a loss of motor drive, as it may signify an adaptive reorganization to maintain motor performance (Peng et al., 2024). Though CMC is widely interpreted as a reflection of descending corticospinal drive (Liu et al., 2019), it is important to recognize that afferent feedback also influences this coupling within the sensorimotor system (Airaksinen et al., 2015). Fatigue-induced changes in peripheral afferent input, particularly from proprioceptive feedback and group III/IV muscle afferents, can modulate cortical excitability and inhibitory circuits (Sidhu et al., 2018), thereby influencing corticospinal output and its synchronization with muscle activity. These afferent-driven mechanisms provide a plausible pathway through which peripheral fatigue contributes to the observed changes in CMC. Fatiguing tasks may induce task-specific adjustments in neural gain and excitability across multiple levels of the corticospinal system, including both cortical and spinal components (Boonstra, 2017; Weavil and Amann, 2018). The corticospinal pathway, considered the primary conduit for voluntary motor control in humans, may exhibit task-specific alterations in excitability during exercise (Weavil and Amann, 2018), reflecting a dynamic balance between excitatory and inhibitory influences at the motor cortex and spinal motoneuron levels. As corticospinal pathways become less effective due to fatigue, the nervous system may reallocate control to subcortical structures such as the basal ganglia and cerebellum, as well as to spinal networks. Functional magnetic resonance imaging studies (Liu et al., 2003; van Duinen et al., 2007; Jiang et al., 2016) have reported increased connectivity between the motor cortex and subcortical structures during fatiguing tasks, suggesting that subcortical regions increasingly support motor performance as primary cortical circuits become fatigued. Additionally, reduced CMC with fatigability is often accompanied by enhanced intermuscular coherence among synergistic muscles (Boonstra et al., 2008), pointing to a greater reliance on spinal-mediated muscle coordination. Spinal interneurons, which organize rhythmic and synergistic muscle activations, provide a more automated mode of control when precise cortical regulation becomes inefficient (Giszter, 2015). These findings challenge the simple assumption that sustained or elevated cortical activity solely supports motor performance under fatigue. Instead, neuromuscular fatigability may trigger a reorganization of the motor control hierarchy, where cortical involvement may increase initially but diminishes as subcortical and spinal mechanisms take over. Future models of neuromuscular fatigability may consider this adaptive redistribution of motor control rather than focusing solely on cortical output escalation. This shift allows the nervous system to preserve motor output through more automated or synergistic control pathways. Therefore, interventions aiming to support motor performance under fatigue (e.g., brain stimulation protocols) can consider not only enhancing cortical activity but also facilitating the transition to efficient downstream (subcortical/spinal) control.

Alpha-band CMC has received limited attention, being examined in only four of the fifteen studies included. A plausible explanation for this may be that alpha oscillations play a more prominent role during movement preparation, attentional engagement, or early task phases (Deiber et al., 2012; Jensen and Bonnefond, 2026), whereas fatigability studies typically focus on steady-state or end-task epochs, potentially overlooking transient alpha-related dynamics.

### 4.2 Upper- vs. lower-limb muscles: should we expect distinct adaptations in neuromuscular fatigability?

​The evidence in this review does not show a consistent difference between upper- and lower-limb muscles in their CMC responses during neuromuscular fatigability. Instead, the reviewed studies reveal substantial within-limb heterogeneity, with CMC adaptations varying as a function of task demands, contraction intensity, muscle group, and stage of neuromuscular fatigue. When considering task design, upper-limb studies were evenly split between sustained (4 studies) and intermittent (4 studies) contractions, including handgrip, elbow flexion/extension, and shoulder abduction, whereas lower-limb studies were dominated by sustained dorsiflexion (5 studies), with only one intermittent knee extension/flexion study and one pedaling task. This distribution indicates that differences in task type may contribute to within-limb variability and could partly explain heterogeneity in CMC outcomes, rather than limb identity alone. From a neurophysiological perspective, limb-dependent differences in fatigability are often attributed to variations in cortical representation, functional role, and relative reliance on corticospinal versus subcortical or spinal pathways (Luft et al., 2002). Several studies highlight distinct neuromuscular fatigability responses between the upper- and lower-limbs’ muscles. Temesi et al (Temesi et al., 2019) and Vernillo et al (Vernillo et al., 2020) demonstrated that sustained voluntary contractions led to reduced motor drive, essential for initiating and sustaining muscle contractions, in the biceps brachii and elbow flexors but remained unchanged in knee extensors, suggesting different recovery patterns between limbs. A reduction in this drive may imply that more effort or compensatory input is required to maintain motor output. In a related study, Vernillo et al (Vernillo et al., 2018) observed greater neuromuscular fatigability in the knee extensors, primarily due to central fatigability, which refers to impairments in the CNS to drive the muscle. In contrast, the elbow flexors exhibited more pronounced peripheral (muscular) fatigability, indicating failure at or beyond the neuromuscular junction or within the muscle itself, along with increased corticospinal inhibition, suggesting a stronger reduction in the excitability of the motor pathways from brain to muscle. Boccia et al (Boccia et al., 2017) reported that during the fatiguing task, the knee extensors exhibited a larger reduction in maximal force output, while the elbow extensors showed a greater decline in the rate of force development. These differences highlight that fatigability affects muscle groups in distinct ways, depending on their functional roles and neuromuscular characteristics. However, such mechanisms do not translate into a straightforward or predictable pattern of CMC modulation across upper- and lower-limbs, where the relationship between neuromuscular fatigability and CMC may be better interpreted in terms of how oscillatory coupling reflects adaptive changes in motor control strategies (Reyes et al., 2017). Consistent with earlier observations, reductions in beta or gamma band CMC with fatiguing contractions may reflect diminished corticospinal synchronization. Importantly, this does not necessarily indicate reduced overall neural drive, but rather a possible redistribution of control toward subcortical and spinal circuits that can support motor output as fatigue develops. While preserved or increased beta band CMC, reported in both upper- and lower-limb tasks, might indicate a compensatory stabilization strategy, whereby the nervous system enhances oscillatory coupling to maintain force output during sustained contractions.

### 4.3 Task characteristics: do they affect brain-muscle communication during neuromuscular fatigability?

The present synthesis indicates that CMC responses with neuromuscular fatigability are shaped by task characteristics, particularly contraction type, mode, and intensity. Although beta band coherence was most often reported to change during fatiguing tasks, the direction and magnitude of these changes varied widely across studies.

Intermittent or block-designed paradigms often yielded reduced or stable block-averaged coherence despite within-block modulation (Hsu et al., 2023; Bedard et al., 2025). Evidence from studies examining fatigability indicates that periodic recovery during intermittent contractions preserves motor unit firing behaviour, maintains reflex excitability, and limits central drive deterioration compared with sustained efforts (Bigland-Ritchie et al., 1986; Christensen and Fuglsang-Frederiksen, 1988; Duchateau et al., 2002). Such recovery phases may permit partial restoration of corticospinal timing precision between blocks. This intermittent re-stabilization of neural drive could counteract progressive desynchronization, thereby masking cumulative declines in brain–muscle coherence (Bakken et al., 2024). Consequently, the absence of net CMC changes in block-based designs may reflect preserved or dynamically restored synchronization. On the other hand, sustained contractions impose continuous metabolic and neural stress, promoting progressive alterations in afferent feedback and corticospinal excitability that may more strongly influence coherence magnitude (Hunter et al., 2016). In low-intensity sustained isometric contractions, increased synchronization likely reflects temporally structured descending input contributing to stable force output (Mima and Hallett, 1999; Conway et al., 2004). During low-intensity sustained tasks, increases in coherence observed in some studies may indicate a reinforcement of temporal coupling within corticospinal circuits as the muscular contractile efficiency declines and greater precision of descending timing is required to maintain force (Peng et al., 2024). However, as fatigue progresses, several mechanisms may weaken this coupling. Accumulation of metabolites activates group III/IV afferents, altering spinal excitability and supraspinal drive (Amann et al., 2015); motor unit discharge becomes more variable (Christie and Kamen, 2009); and the intracortical inhibitory–excitatory balance is modified (Hunter et al., 2016). Each of these factors can degrade the temporal alignment necessary for strong oscillatory synchronization, potentially explaining reductions in coherence during later stages of fatigue. At higher contraction intensities or during tasks performed to failure, coherence responses were more frequently attenuated or transiently broadened. Strong contractions recruit larger motor units (Milner-Brown et al., 1973) and increase overall cortical activation (Wang et al., 2023), yet an increased drive may not necessarily translate into stronger oscillatory synchronization (Boonstra, 2017). Experimental work, along with findings from included studies (Igasaki et al., 2018; Igasaki et al., 2019), has shown that high-force outputs can reduce beta-range synchronization despite elevated motor cortex activity (Andrykiewicz et al., 2007), suggesting that intense descending input may alter the spectral distribution of corticospinal signals (from beta band CMC to gamma band CMC) rather than reinforce specific-band rhythmic coupling. As fatigue increases under these conditions, reductions in voluntary activation and altered corticospinal excitability may further compromise the stability of oscillatory transmission along descending pathways.

### 4.4 Future Research: toward terminological and methodological consistency in neuromuscular fatigability research

One major barrier to synthesizing findings across studies is the inconsistent and often unclear use of terminology and assessment timepoints related to CMC and neuromuscular fatigability. Terms such as “rest,” “pre-fatigue,” and “post-fatigue” are frequently used without standardized definitions. For example, “rest” may refer either to a true baseline prior to movement or to low-intensity contractions performed before higher levels of fatigue have developed. Similarly, “post-fatigue” may capture a wide range of physiological states, from early signs of fatigue to severe exhaustion or even recovery, making it difficult to interpret what phase of fatigue is being assessed. An additional challenge to this is the variation in how studies divide time periods for analysis (epochs). Some studies, such as Ushijima et al (Ushijima et al., 2017) and Igasaki et al (Igasaki et al., 2018; Igasaki et al., 2019), divide contraction periods into fine-grained epochs (e.g., 12-second segments), while others use broader stages, e.g., early vs. late fatigue in Yang et al (Yang et al., 2009) or block-level analysis in Yang et al (Yang et al., 2010). Others still, like Wang et al (Wang et al., 2020), rely on a simpler pre- vs. post-fatigue comparison, which makes it harder to capture the dynamic progression of neuromuscular fatigability. Without a standardized framework for defining fatigue phases and coherence measurement windows, cross-study comparisons remain difficult, and conclusions about the temporal evolution of CMC with fatigability may be misinterpreted. Future research should adopt consistent terminology and explicitly report the criteria used to define fatigue states and segment coherence data, facilitating better and more interpretable analyses. Furthermore, future studies could complement coherence analysis by employing non-invasive brain stimulation (NIBS) techniques, such as transcranial magnetic stimulation (TMS) or transcranial alternating current stimulation (tACS), to investigate the causal role of cortical excitability and rhythmic activity in neuromuscular fatigue. These approaches may help clarify whether observed CMC changes reflect central mechanisms of fatigue or compensatory processes, offering a deeper understanding of the neurophysiological mechanisms involved.

### 4.5 Limitations

A major limitation of the current review is the scarcity of studies conducted in neurological populations. Despite the clear clinical relevance of neuromuscular fatigability in conditions such as stroke, multiple sclerosis, and spinal cord injury, and the importance of CMC as a marker of corticospinal communication, only one included study involved participants with a neurological disorder (cerebral palsy). Importantly, this scarcity is not only due to a lack of research but also reflects specific methodological mismatches between existing clinical studies and the inclusion criteria of this review. Many studies in neurological populations were excluded because they did not employ experimentally induced neuromuscular fatigability (e.g., focusing instead on perceived or trait fatigue), did not include concurrent EEG–EMG recordings required to assess CMC, or did not quantify fatigue-related changes within a defined pre–post or during-fatigue framework. In addition, some studies focused on resting-state or non-fatiguing paradigms, which do not capture state-dependent changes in corticospinal coupling. As a result, most of the evidence summarized in this review is derived from studies conducted on healthy individuals. Although these findings provide valuable insights into the neurophysiological mechanisms underlying fatigability, their direct generalization to neurological populations is not possible. The scarcity of such studies likely reflects ethical constraints and practical challenges of inducing fatigue during experimental tasks in clinical populations, particularly those with pathological fatigue or progressive neuromuscular decline (e.g., multiple sclerosis), limiting the implementation of high-intensity fatiguing protocols. These challenges may arise from several factors, including reduced task tolerance due to motor impairment, variability related to lesion location and disease severity, and neurological deficits such as spasticity, impaired sensory feedback, and altered corticospinal integrity, all of which may independently affect EEG, EMG, and CMC measures. Furthermore, comorbidities, medication use, and cognitive impairments may further complicate experimental design and interpretation of fatigue-related neurophysiological changes in these populations. While the limited neurological evidence in this review precludes condition-specific recommendations for task/study design, the methodological heterogeneity identified across included studies allows us to propose a minimum reporting and design framework applicable across populations. Future studies should consider standardizing task characteristics (e.g., contraction type, intensity, and duration), clearly defining fatigue criteria, phases, assessment time points, and coherence computation methods to improve reproducibility and facilitate cross-study comparisons.

This review has several other limitations that also highlight key areas for future research. Namely, the 15 included studies exhibited substantial methodological heterogeneity, including task type, intensity, contraction mode, targeted muscle(s) and limbs, assessment timepoint, and coherence analysis methods, which limits comparability across studies. Second, inconsistent terminology (e.g., varying definitions of “pre-fatigue” and “post-fatigue”) complicates data synthesis and should be consistently adopted and clarified in future work. Finally, the existing literature is biased toward young to middle-aged adults (29.9 ± 5.9 years), despite evidence that aging independently alters CMC, leaving age-related differences in fatigue-induced CMC alterations unexplored (da Silva Costa et al., 2024). Addressing these gaps may not only enhance the understanding of fatigue mechanisms but also inform targeted interventions in both healthy and clinical populations.

## 5 Conclusion

Across the 15 included studies, beta band CMC emerged as the most consistently reported and interpretable frequency range, yet its modulation with neuromuscular fatigability was not uniform. Transient increases in beta coherence were observed during early or within-task phases in some paradigms, whereas progressive reductions were more commonly reported during prolonged, high-intensity, or later stages of fatigue. Alpha band CMC was infrequently examined and showed minimal or inconsistent modulation. Gamma band CMC often appeared transiently during task initiation or high-force conditions and diminished as fatigue accumulated. Collectively, these findings indicate that fatigue-related changes in CMC are context-dependent rather than stereotyped and should not be interpreted as a simple proxy for loss of motor drive. This review advances the literature by showing that CMC modulation with fatigability may depend on when fatigue is assessed, how CMC is analyzed, and the characteristics of the fatiguing task. The synthesis also identifies important methodological and conceptual gaps, including inconsistent definitions of fatigue-related states, limited use of time-resolved analyses, and a near-absence of studies in neurological populations (only one study in cerebral palsy).

## References

[B25] Abd-Elfattah H. M. Abdelazeim F. H. Elshennawy S. (2015). Physical and cognitive consequences of fatigue: A review. J. Advanced Res. 6, 351–358. doi: 10.1016/j.jare.2015.01.011, PMID: 26257932 PMC4522584

[B30] Airaksinen K. Lehti T. Nurminen J. Luoma J. Helle L. Taulu S. . (2015). Cortico-muscular coherence parallels coherence of postural tremor and MEG during static muscle contraction. Neurosci. Lett. 602, 22–26. doi: 10.1016/j.neulet.2015.06.034, PMID: 26116820

[B95] Amann M. Sidhu S. K. Weavil J. C. Mangum T. S. Venturelli M. (2015). Autonomic responses to exercise: group III/IV muscle afferents and fatigue. Auton Neurosci. 188, 19–23. doi: 10.1016/j.autneu.2014.10.018, PMID: 25458423 PMC4336599

[B99] Andrykiewicz A. Patino L. Naranjo J. R. Witte M. Hepp-Reymond M.-C. Kristeva R. (2007). Corticomuscular synchronization with small and large dynamic force output. BMC Neurosci. 8, 101. doi: 10.1186/1471-2202-8-101, PMID: 18042289 PMC2245954

[B19] Anton H. A. Miller W. C. Townson A. F. (2008). Measuring fatigue in persons with spinal cord injury. Arch. Phys. Med. Rehabil. 89, 538–542. doi: 10.1016/j.apmr.2007.11.009, PMID: 18295634 PMC3595300

[B22] Anton H. A. Miller W. C. Townson A. F. Imam B. Silverberg N. Forwell S. (2017). The course of fatigue after acute spinal cord injury. Spinal Cord. 55, 94–97. doi: 10.1038/sc.2016.102, PMID: 27349608

[B92] Bakken K. Horton C. Fisher M. Wadsley C. G. Greenhouse I. (2024). Corticospinal excitability at rest outside of a task does not differ from task intertrial intervals in healthy adults. Exp. Brain Res. 242, 2263–2270. doi: 10.1007/s00221-024-06895-8, PMID: 39043898 PMC11759664

[B55] Bedard P. Knutson K. M. McGurrin P. M. Vial F. Popa T. Horovitz S. G. . (2025). Multimodal neuroimaging of fatigability development. Imaging Neurosci. (Camb) 3. doi: 10.1162/IMAG.a.132, PMID: 40909358 PMC12406056

[B89] Bigland-Ritchie B. Furbush F. Woods J. J. (1986). Fatigue of intermittent submaximal voluntary contractions: central and peripheral factors. J. Appl. Physiol. (1985). 61, 421–429. doi: 10.1152/jappl.1986.61.2.421, PMID: 3745035

[B8] Bizid R. Margnes E. François Y. Jully J. L. Gonzalez G. Dupui P. . (2009). Effects of knee and ankle muscle fatigue on postural control in the unipedal stance. Eur. J. Appl. Physiol. 106, 375–380. doi: 10.1007/s00421-009-1029-2, PMID: 19288126

[B88] Boccia G. Dardanello D. Zoppirolli C. Bortolan L. Cescon C. Schneebeli A. . (2017). Central and peripheral fatigue in knee and elbow extensor muscles after a long-distance cross-country ski race. Scand. J. Med. Sci. Sports. 27, 945–955. doi: 10.1111/sms.12718, PMID: 27293016

[B75] Boonstra T. W. (2017). Cortical adaptations during muscle fatigue: the role of sensorimotor oscillations. Acta Physiologica. 220, 307–309. doi: 10.1111/apha.12879, PMID: 28370992

[B80] Boonstra T. W. Daffertshofer A. van Ditshuizen J. C. van den Heuvel M. R. Hofman C. Willigenburg N. W. . (2008). Fatigue-related changes in motor-unit synchronization of quadriceps muscles within and across legs. J. Electromyogr Kinesiol. 18, 717–731. doi: 10.1016/j.jelekin.2007.03.005, PMID: 17462912

[B72] Buzsáki G. Wang X. J. (2012). Mechanisms of gamma oscillations. Annu. Rev. Neurosci. 35, 203–225. doi: 10.1146/annurev-neuro-062111-150444, PMID: 22443509 PMC4049541

[B3] Carroll T. J. Taylor J. L. Gandevia S. C. (2017). Recovery of central and peripheral neuromuscular fatigue after exercise. J. Appl. Physiol. 122, 1068–1076. doi: 10.1152/japplphysiol.00775.2016, PMID: 27932676

[B26] Casamento-Moran A. Mooney R. A. Chib V. S. Celnik P. A. (2023). Cerebellar excitability regulates physical fatigue perception. J. Neurosci. 43, 3094. doi: 10.1523/JNEUROSCI.1406-22.2023, PMID: 36914263 PMC10146467

[B90] Christensen H. Fuglsang-Frederiksen A. (1988). Quantitative surface EMG during sustained and intermittent submaximal contractions. Electroencephalogr Clin. Neurophysiol. 70, 239–247. doi: 10.1016/0013-4694(88)90084-3, PMID: 2458230

[B96] Christie A. Kamen G. (2009). Motor unit firing behavior during prolonged 50% MVC dorsiflexion contractions in young and older adults. J. Electromyography Kinesiology. 19, 543–552. doi: 10.1016/j.jelekin.2008.03.005, PMID: 18448360

[B94] Conway B. A. Reid C. Halliday D. M. (2004). Low frequency corlico-muscular coherence during voluntary rapid movements of the wrist joint. Brain Topogr. 16, 221–224. doi: 10.1023/b:brat.0000032855.99865.31, PMID: 15379217

[B12] Corcos D. M. Jiang H. Y. Wilding J. Gottlieb G. L. (2002). Fatigue induced changes in phasic muscle activation patterns for fast elbow flexion movements. Exp. Brain Res. 142, 1–12. doi: 10.1007/s00221-001-0904-9, PMID: 11797079

[B54] Correia J. P. Domingos C. Witvrouw E. Luís P. Rosa A. Vaz J. R. . (2024). Brain and muscle activity during fatiguing maximum-speed knee movement. J. Appl. Physiol. (1985). 136, 200–212. doi: 10.1152/japplphysiol.00145.2023, PMID: 38059285

[B100] da Silva Costa A. A. Moraes R. den Otter R. Gennaro F. Bakker L. Rocha Dos Santos P. C. . (2024). Corticomuscular and intermuscular coherence as a function of age and walking balance difficulty. Neurobiol. Aging. 141, 85–101. doi: 10.1016/j.neurobiolaging.2024.05.004, PMID: 38850592

[B82] Deiber M.-P. Sallard E. Ludwig C. Ghezzi C. Barral J. Ibanez V. (2012). EEG alpha activity reflects motor preparation rather than the mode of action selection. Front. Integr. Neurosci. 6. doi: 10.3389/fnint.2012.00059, PMID: 22912607 PMC3418545

[B23] Dobkin B. H. (2008). Fatigue versus activity-dependent fatigability in patients with central or peripheral motor impairments. Neurorehabil Neural Repair. 22, 105–110. doi: 10.1177/1545968308315046, PMID: 18285599 PMC4160309

[B91] Duchateau J. Balestra C. Carpentier A. Hainaut K. (2002). Reflex regulation during sustained and intermittent submaximal contractions in humans. J. Physiol. 541, 959–967. doi: 10.1113/jphysiol.2002.016790, PMID: 12068054 PMC2290373

[B64] Engel A. K. Fries P. (2010). Beta-band oscillations--signalling the status quo? Curr. Opin. Neurobiol. 20, 156–165. doi: 10.1016/j.conb.2010.02.015, PMID: 20359884

[B58] Enoka R. M. Duchateau J. (2016). Translating fatigue to human performance. Med. Sci. Sports Exerc. 48, 2228–2238. doi: 10.1249/MSS.0000000000000929, PMID: 27015386 PMC5035715

[B32] Fang Y. Daly J. J. Sun J. Hvorat K. Fredrickson E. Pundik S. . (2009). Functional corticomuscular connection during reaching is weakened following stroke. Clin. Neurophysiol. 120, 994–1002. doi: 10.1016/j.clinph.2009.02.173, PMID: 19362515 PMC2680928

[B51] Forman C. R. Jacobsen K. J. Karabanov A. N. Nielsen J. B. Lorentzen J. (2022). Corticomuscular coherence is reduced in relation to dorsiflexion fatigability to the same extent in adults with cerebral palsy as in neurologically intact adults. Eur. J. Appl. Physiol. 122, 1459–1471. doi: 10.1007/s00421-022-04938-y, PMID: 35366090

[B37] Gao Z. Lv S. Ran X. Wang Y. Xia M. Wang J. . (2024). Influencing factors of corticomuscular coherence in stroke patients. Front. Hum. Neurosci. 18. doi: 10.3389/fnhum.2024.1354332, PMID: 38562230 PMC10982423

[B15] Gates D. H. Dingwell J. B. (2008). The effects of neuromuscular fatigue on task performance during repetitive goal-directed movements. Exp. Brain Res. 187, 573–585. doi: 10.1007/s00221-008-1326-8, PMID: 18327575 PMC2825378

[B27] Gerloff C. Braun C. Staudt M. Hegner Y. L. Dichgans J. Krägeloh-Mann I. (2006). Coherent corticomuscular oscillations originate from primary motor cortex: evidence from patients with early brain lesions. Hum. Brain Mapp. 27, 789–798. doi: 10.1002/hbm.20220, PMID: 16475178 PMC6871432

[B81] Giszter S. F. (2015). Motor primitives--new data and future questions. Curr. Opin. Neurobiol. 33, 156–165. doi: 10.1016/j.conb.2015.04.004, PMID: 25912883 PMC6524953

[B9] Gribble P. A. Hertel J. (2004). Effect of lower-extremity muscle fatigue on postural control. Arch. Phys. Med. Rehabil. 85, 589–592. doi: 10.1016/j.apmr.2003.06.031, PMID: 15083434

[B70] Gwin J. T. Ferris D. P. (2012). Beta- and gamma-range human lower limb corticomuscular coherence. Front. Hum. Neurosci. 6, 258. doi: 10.3389/fnhum.2012.00258, PMID: 22973219 PMC3438504

[B71] He B. J. (2014). Scale-free brain activity: past, present, and future. Trends Cognit. Sci. 18, 480–487. doi: 10.1016/j.tics.2014.04.003, PMID: 24788139 PMC4149861

[B52] Hsu L.-I. Lim K.-W. Lai Y.-H. Chen C.-S. Chou L.-W. (2023). Effects of muscle fatigue and recovery on the neuromuscular network after an intermittent handgrip fatigue task: spectral analysis of electroencephalography and electromyography signals. Sensors. 23, 2440. doi: 10.3390/s23052440, PMID: 36904641 PMC10007140

[B93] Hunter S. K. McNeil C. J. Butler J. E. Gandevia S. C. Taylor J. L. (2016). Short-interval cortical inhibition and intracortical facilitation during submaximal voluntary contractions changes with fatigue. Exp. Brain Res. 234, 2541–2551. doi: 10.1007/s00221-016-4658-9, PMID: 27165508 PMC5349854

[B47] Igasaki T. Yamashita K. Ushijima T. (2018). “ Force-temporal characteristics of EEG-EMG coherence during isometric contraction of the tibialis anterior muscle”, in: 2018 11th Biomedical Engineering International Conference (BMEiCON) (US & Canada: Institute of Electrical and Electronic Engineers). 10.1109/EMBC.2019.8856456 31946328

[B50] Igasaki T. Yamashita K. Ushijima T. (2019). Force-temporal characteristics of EEG-EMG coherence during isometric contraction of lateral head of gastrocnemius muscle. Annu. Int. Conf IEEE Eng. Med. Biol. Soc 2019, 2157–2160. doi: 10.1109/EMBC.2019.8856456, PMID: 31946328

[B14] Jaric S. Blesic S. Milanovic S. Radovanovic S. Ljubisavljevic M. Anastasijevic R. (1999). Changes in movement final position associated with agonist and antagonist muscle fatigue. Eur. J. Appl. Physiol. Occup. Physiol. 80, 467–471. doi: 10.1007/s004210050619, PMID: 10502081

[B83] Jensen O. Bonnefond M. (2026). The alpha rhythm: from physiology to behaviour. Physiol. Rev. doi: 10.1152/physrev.00001.2025, PMID: 41633382 PMC7618721

[B77] Jiang Z. Wang X. F. Yue G. H. (2016). Strengthened corticosubcortical functional connectivity during muscle fatigue. Neural Plast. 2016, 1726848. doi: 10.1155/2016/1726848, PMID: 27830093 PMC5086541

[B5] Juárez-Belaúnde A. Orcajo E. Lejarreta S. Davila-Pérez P. León N. Oliviero A. (2021). Fatigue in patients with acquired brain damage (Spain: Neurologia (Engl Ed). 10.1016/j.nrleng.2024.01.008 38278413

[B21] Kotwani S. Gadgil S. Ranade P. (2018). Comparison of neuromuscular fatigue in chronic stroke patients with healthy controls. Clin. Invest. 08. doi: 10.4172/Clinical-Investigation.1000140

[B2] Kratz A. L. Murphy S. L. Braley T. J. Basu N. Kulkarni S. Russell J. . (2019). Development of a person-centered conceptual model of perceived fatigability. Qual Life Res. 28, 1337–1347. doi: 10.1007/s11136-018-2093-z, PMID: 30604341 PMC7395299

[B79] Liu J. Z. Shan Z. Y. Zhang L. D. Sahgal V. Brown R. W. Yue G. H. (2003). Human brain activation during sustained and intermittent submaximal fatigue muscle contractions: an FMRI study. J. Neurophysiol. 90, 300–312. doi: 10.1152/jn.00821.2002, PMID: 12634278

[B34] Liu J. Sheng Y. Liu H. (2019). Corticomuscular coherence and its applications: A review. Front. Hum. Neurosci. 13. doi: 10.3389/fnhum.2019.00100, PMID: 30949041 PMC6435838

[B13] Lorist M. M. Kernell D. Meijman T. F. Zijdewind I. (2002). Motor fatigue and cognitive task performance in humans. J. Physiol. 545, 313–319. doi: 10.1113/jphysiol.2002.027938, PMID: 12433971 PMC2290666

[B84] Luft A. R. Smith G. V. Forrester L. Whitall J. Macko R. F. Hauser T. K. . (2002). Comparing brain activation associated with isolated upper and lower limb movement across corresponding joints. Hum. Brain Mapp. 17, 131–140. doi: 10.1002/hbm.10058, PMID: 12353246 PMC6872124

[B60] Maeo S. Balshaw T. G. Lanza M. B. Hannah R. Folland J. P. (2021). Corticospinal excitability and motor representation after long-term resistance training. Eur. J. Neurosci. 53, 3416–3432. doi: 10.1111/ejn.15197, PMID: 33763908

[B53] Magnuson J. R. Bruce C. D. Dalton B. H. McNeil C. J. (2024). Oscillatory drive is increased and steadiness is impaired when torque but not EMG is matched during a fatiguing contraction. J. Neurophysiol. 132, 1907–1916. doi: 10.1152/jn.00309.2024, PMID: 39503585

[B48] Martínez-Aguilar G. M. Gutiérrez D. (2019). Using cortico-muscular and cortico-cardiac coherence to study the role of the brain in the development of muscular fatigue. Biomed. Signal Process. Control. 48, 153–160. doi: 10.1016/j.bspc.2018.10.011, PMID: 38826717

[B31] Maura R. M. Rueda Parra S. Stevens R. E. Weeks D. L. Wolbrecht E. T. Perry J. C. (2023). Literature review of stroke assessment for upper-extremity physical function via EEG, EMG, kinematic, and kinetic measurements and their reliability. J. Neuroeng Rehabil. 20, 21. doi: 10.1186/s12984-023-01142-7, PMID: 36793077 PMC9930366

[B66] McManus L. Hu X. Rymer W. Z. Suresh N. L. Lowery M. M. (2016). Muscle fatigue increases beta-band coherence between the firing times of simultaneously active motor units in the first dorsal interosseous muscle. J. Neurophysiol. 115, 2830–2839. doi: 10.1152/jn.00097.2016, PMID: 26984420 PMC4922605

[B68] Meng F. Tong K. Y. Chan S. T. Wong W. W. Lui K. H. Tang K. W. . (2009). Cerebral plasticity after subcortical stroke as revealed by cortico-muscular coherence. IEEE Trans. Neural Syst. Rehabil. Eng. 17, 234–243. doi: 10.1109/TNSRE.2008.2006209, PMID: 19273041

[B97] Milner-Brown H. S. Stein R. B. Yemm R. (1973). The orderly recruitment of human motor units during voluntary isometric contractions. J. Physiol. 230, 359–370. doi: 10.1113/jphysiol.1973.sp010192, PMID: 4350770 PMC1350367

[B69] Mima T. Hallett M. (1999). Electroencephalographic analysis of cortico-muscular coherence: reference effect, volume conduction and generator mechanism. Clin. Neurophysiology. 110, 1892–1899. doi: 10.1016/S1388-2457(99)00238-2, PMID: 10576484

[B56] Moher D. Liberati A. Tetzlaff J. Altman D. G. (2009). Preferred reporting items for systematic reviews and meta-analyses: the PRISMA statement. PloS Med. 6, e1000097. doi: 10.1371/journal.pmed.1000097, PMID: 19621072 PMC2707599

[B59] Moscatelli F. Messina G. Valenzano A. Petito A. Triggiani A. I. Messina A. . (2016). Differences in corticospinal system activity and reaction response between karate athletes and non-athletes. Neurol. Sci. 37, 1947–1953. doi: 10.1007/s10072-016-2693-8, PMID: 27544220

[B17] Nardon M. Sinha O. Kpankpa J. Albenze E. Bonnet C. Bertucco M. . (2024). Prioritized adjustments in posture stabilization and adaptive reaching during neuromuscular fatigue of lower-limb muscles. bioRxiv. doi: 10.1152/japplphysiol.00252.2024, PMID: 39024408

[B63] Newson J. J. Thiagarajan T. C. (2019). EEG frequency bands in psychiatric disorders: A review of resting state studies. Front. Hum. Neurosci. 12. doi: 10.3389/fnhum.2018.00521, PMID: 30687041 PMC6333694

[B35] Omlor W. Patino L. Mendez-Balbuena I. Schulte-Mönting J. Kristeva R. (2011). Corticospinal beta-range coherence is highly dependent on the pre-stationary motor state. J. Neurosci. 31, 8037–8045. doi: 10.1523/JNEUROSCI.4153-10.2011, PMID: 21632925 PMC6622845

[B57] Ouzzani M. Hammady H. Fedorowicz Z. Elmagarmid A. (2016). Rayyan—a web and mobile app for systematic reviews. Systematic Rev. 5, 210. doi: 10.1186/s13643-016-0384-4, PMID: 27919275 PMC5139140

[B24] Patejdl R. Zettl U. K. (2022). The pathophysiology of motor fatigue and fatigability in multiple sclerosis. Front. Neurol. 13. doi: 10.3389/fneur.2022.891415, PMID: 35968278 PMC9363784

[B73] Peng J. Zikereya T. Shao Z. Shi K. (2024). The neuromechanical of Beta-band corticomuscular coupling within the human motor system. Front. Neurosci. 18, 1441002. doi: 10.3389/fnins.2024.1441002, PMID: 39211436 PMC11358111

[B16] Pethick J. Tallent J. (2022). The neuromuscular fatigue-induced loss of muscle force control. Sports. 10, 184. doi: 10.3390/sports10110184, PMID: 36422953 PMC9694672

[B33] Pichiorri F. Toppi J. de Seta V. Colamarino E. Masciullo M. Tamburella F. . (2023). Exploring high-density corticomuscular networks after stroke to enable a hybrid Brain-Computer Interface for hand motor rehabilitation. J. Neuroeng Rehabil. 20, 5. doi: 10.1186/s12984-023-01127-6, PMID: 36639665 PMC9840279

[B40] Pollok B. Krause V. Martsch W. Wach C. Schnitzler A. Südmeyer M. (2012). Motor-cortical oscillations in early stages of Parkinson’s disease. J. Physiol. 590, 3203–3212. doi: 10.1113/jphysiol.2012.231316, PMID: 22547636 PMC3406400

[B38] Proudfoot M. van Ede F. Quinn A. Colclough G. L. Wuu J. Talbot K. . (2018). Impaired corticomuscular and interhemispheric cortical beta oscillation coupling in amyotrophic lateral sclerosis. Clin. Neurophysiol. 129, 1479–1489. doi: 10.1016/j.clinph.2018.03.019, PMID: 29678369

[B46] Qiu Q. Cao L. Hao D. Yang L. Hillstrom R. Zheng D. (2018). Muscle extremely low frequency magnetic stimulation eliminates the effect of fatigue on EEG-EMG coherence during the lateral raise task: A pilot quantitative investigation. BioMed. Res. Int. 2018, 7673068. doi: 10.1155/2018/7673068, PMID: 30079351 PMC6069696

[B65] Reyes A. Laine C. M. Kutch J. J. Valero-Cuevas F. J. (2017). Beta band corticomuscular drive reflects muscle coordination strategies. Front. Comput. Neurosci. 11. doi: 10.3389/fncom.2017.00017, PMID: 28420975 PMC5378725

[B20] Romani A. (2008). The treatment of fatigue. Neurol. Sci. 29 Suppl 2, S247–S249. doi: 10.1007/s10072-008-0952-z, PMID: 18690507

[B6] Royer N. Coates K. Aboodarda S. J. Camdessanché J. P. Millet G. Y. (2022). How is neuromuscular fatigability affected by perceived fatigue and disability in people with multiple sclerosis? Front. Neurol. 13, 983643. doi: 10.3389/fneur.2022.983643, PMID: 36324385 PMC9618894

[B10] Salavati M. Moghadam M. Ebrahimi I. Arab A. M. (2007). Changes in postural stability with fatigue of lower extremity frontal and sagittal plane movers. Gait Posture. 26, 214–218. doi: 10.1016/j.gaitpost.2006.09.001, PMID: 17049237

[B67] Salomon I. (2024). Neurobiological insights into cerebral palsy: A review of the mechanisms and therapeutic strategies. Brain Behavior. 14, e70065. doi: 10.1002/brb3.70065, PMID: 39378294 PMC11460637

[B74] Sidhu S. K. Weavil J. C. Thurston T. S. Rosenberger D. Jessop J. E. Wang E. . (2018). Fatigue-related group III/IV muscle afferent feedback facilitates intracortical inhibition during locomotor exercise. J. Physiol. 596, 4789–4801. doi: 10.1113/JP276460, PMID: 30095164 PMC6166070

[B61] Škarabot J. Casolo A. Balshaw T. G. Maeo S. Lanza M. B. Holobar A. . (2024). Greater motor unit discharge rate during rapid contractions in chronically strength-trained individuals. J. Neurophysiol. 132, 1896–1906. doi: 10.1152/jn.00017.2024, PMID: 39527019 PMC11687832

[B18] Søgaard K. Gandevia S. C. Todd G. Petersen N. T. Taylor J. L. (2006). The effect of sustained low-intensity contractions on supraspinal fatigue in human elbow flexor muscles. J. Physiol. 573, 511–523. doi: 10.1113/jphysiol.2005.103598, PMID: 16556656 PMC1779725

[B62] Sterne J. A. C. Hernán M. A. Reeves B. C. Savović J. Berkman N. D. Viswanathan M. . (2016). ROBINS-I: a tool for assessing risk of bias in non-randomised studies of interventions. BMJ 355, i4919. doi: 10.1136/bmj.i4919, PMID: 27733354 PMC5062054

[B36] Stokkermans M. Solis-Escalante T. Cohen M. X. Weerdesteyn V. (2023). Distinct cortico-muscular coupling between step and stance leg during reactive stepping responses. Front. Neurol. 14. doi: 10.3389/fneur.2023.1124773, PMID: 36998772 PMC10043329

[B29] Tecchio F. Melgari J. M. Zappasodi F. Porcaro C. Milazzo D. Cassetta E. . (2008). Sensorimotor integration in focal task-specific hand dystonia: a magnetoencephalographic assessment. Neuroscience. 154, 563–571. doi: 10.1016/j.neuroscience.2008.03.045, PMID: 18472344

[B28] Tecchio F. Zappasodi F. Melgari J. M. Porcaro C. Cassetta E. Rossini P. M. (2006). Sensory-motor interaction in primary hand cortical areas: A magnetoencephalography assessment. Neuroscience. 141, 533–542. doi: 10.1016/j.neuroscience.2006.03.059, PMID: 16713107

[B85] Temesi J. Vernillo G. Martin M. Krüger R. L. McNeil C. J. Millet G. Y. (2019). Sustained maximal voluntary contractions elicit different neurophysiological responses in upper- and lower-limb muscles in men. Neuroscience. 422, 88–98. doi: 10.1016/j.neuroscience.2019.09.029, PMID: 31682821

[B41] Tuncel D. Dizibuyuk A. Kiymik M. K. (2010). Time frequency based coherence analysis between EEG and EMG activities in fatigue duration. J. Med. Syst. 34, 131–138. doi: 10.1007/s10916-008-9224-y, PMID: 20433051

[B45] Ushijima T. Sahroni A. Igasaki T. Murayama N. (2017). “ Time-lapse changes in EEG-EMG coherence during weak voluntary contraction of the tibialis anterior muscle”, in: 2017 10th Biomedical Engineering International Conference (BMEiCON) (US & Canada: Institute of Electrical and Electronic Engineers), 2017–2031.

[B44] Ushiyama J. Katsu M. Masakado Y. Kimura A. Liu M. Ushiba J. (2011). Muscle fatigue-induced enhancement of corticomuscular coherence following sustained submaximal isometric contraction of the tibialis anterior muscle. J. Appl. Physiol. (1985). 110, 1233–1240. doi: 10.1152/japplphysiol.01194.2010, PMID: 21393470

[B78] van Duinen H. Renken R. Maurits N. Zijdewind I. (2007). Effects of motor fatigue on human brain activity, an fMRI study. NeuroImage. 35, 1438–1449. doi: 10.1016/j.neuroimage.2007.02.008, PMID: 17408974

[B1] Van Geel F. Moumdjian L. Lamers I. Bielen H. Feys P. (2020). Measuring walking-related performance fatigability in clinical practice: a systematic review. Eur. J. Phys. Rehabil. Med. 56, 88–103. doi: 10.23736/S1973-9087.19.05878-7, PMID: 31742368

[B86] Vernillo G. Temesi J. Martin M. Krüger R. L. Millet G. Y. (2020). Spinal contribution to neuromuscular recovery differs between elbow-flexor and knee-extensor muscles after a maximal sustained fatiguing task. J. Neurophysiol. 124, 763–773. doi: 10.1152/jn.00273.2020, PMID: 32755359

[B87] Vernillo G. Temesi J. Martin M. Millet G. Y. (2018). Mechanisms of fatigue and recovery in upper versus lower limbs in men. Med. Sci. Sports Exerc. 50, 334–343. doi: 10.1249/MSS.0000000000001445, PMID: 28991037

[B39] von Carlowitz-Ghori K. Bayraktaroglu Z. Hohlefeld F. U. Losch F. Curio G. Nikulin V. V. (2014). Corticomuscular coherence in acute and chronic stroke. Clin. Neurophysiol. 125, 1182–1191. doi: 10.1016/j.clinph.2013.11.006, PMID: 24315544

[B7] Vuillerme N. Burdet C. Isableu B. Demetz S. (2006). The magnitude of the effect of calf muscles fatigue on postural control during bipedal quiet standing with vision depends on the eye-visual target distance. Gait Posture. 24, 169–172. doi: 10.1016/j.gaitpost.2005.07.011, PMID: 16226030

[B98] Wang X. Luo Z. Zhang M. Zhao W. Xie S. Wong S. F. . (2023). The interaction between changes of muscle activation and cortical network dynamics during isometric elbow contraction: a sEMG and fNIRS study. Front. Bioeng Biotechnol. 11, 1176054. doi: 10.3389/fbioe.2023.1176054, PMID: 37180038 PMC10167054

[B49] Wang L. Xie Z. Lu A. Lu T. Zhang S. Zheng F. . (2020). Antagonistic muscle prefatigue weakens the functional corticomuscular coupling during isometric elbow extension contraction. Neuroreport. 31, 372–380. doi: 10.1097/WNR.0000000000001387, PMID: 31876688

[B76] Weavil J. C. Amann M. (2018). Corticospinal excitability during fatiguing whole body exercise. Prog. Brain Res. 240, 219–246. doi: 10.1016/bs.pbr.2018.07.011, PMID: 30390833 PMC6363483

[B4] Welch K. A. Kutlubaev M. A. (2020). “ The impact of fatigue on neurorehabilitation,” in Oxford textbook of neurorehabilitation, vol. p . Eds. Dietz V. Ward N. S. Kennard C. Dietz V. Ward N. S. (England Oxford University Press), 0.

[B11] Wilder D. G. Aleksiev A. R. Magnusson M. L. Pope M. H. Spratt K. F. Goel V. K. (1996). Muscular response to sudden load. A tool to evaluate fatigue and rehabilitation. Spine (Phila Pa 1976). 21, 2628–2639. doi: 10.1097/00007632-199611150-00013, PMID: 9045348

[B42] Yang Q. Fang Y. Sun C. K. Siemionow V. Ranganathan V. K. Khoshknabi D. . (2009). Weakening of functional corticomuscular coupling during muscle fatigue. Brain Res. 1250, 101–112. doi: 10.1016/j.brainres.2008.10.074, PMID: 19028460 PMC2655124

[B43] Yang Q. Siemionow V. Yao W. X. Sahgal V. Yue G. H. (2010). Single-trial EEG-EMG coherence analysis reveals muscle fatigue-related progressive alterations in corticomuscular coupling. IEEE Trans. Neural Syst. Rehabil. Engineering. 18, 97–106. doi: 10.1109/TNSRE.2010.2047173, PMID: 20371421

