# Supplementary material for: Dynamics of brain-muscle interaction with neuromuscular fatigability: systematic review
Source: Front Physiol. 2026 Apr 20;17:1719722. doi: 10.3389/fphys.2026.1719722 (PMC13135960; doi:10.3389/fphys.2026.1719722)
Supplement: Supplementary file 1 [file DataSheet1.docx]

# Supplementary Material

1.PubMed:

(("electroencephalography"[MeSH Terms] OR "electroencephalography"[All Fields] OR "eeg"[All Fields] OR "neural correlates"[All Fields] OR "neural activity"[All Fields] OR ("evoked potentials"[MeSH Terms] OR ("evoked"[All Fields] AND "potentials"[All Fields]) OR "evoked potentials"[All Fields] OR "erp"[All Fields]) OR "event-related potentials"[All Fields] OR "spectral power"[All Fields] OR "cortical coherence"[All Fields] OR ("coherence"[All Fields] OR "coherences"[All Fields] OR "coherencies"[All Fields] OR "coherency"[All Fields] OR "coherent"[All Fields] OR "coherently"[All Fields]) OR "frequency bands"[All Fields] OR ("brain waves"[MeSH Terms] OR ("brain"[All Fields] AND "waves"[All Fields]) OR "brain waves"[All Fields] OR "brainwave"[All Fields] OR "brainwaves"[All Fields]) OR ("neurophysiologic"[All Fields] OR "neurophysiological"[All Fields] OR "neurophysiologically"[All Fields]) OR ("neurophysiology"[MeSH Terms] OR "neurophysiology"[All Fields]) OR "brain oscillations"[All Fields])

AND

("fatiguability"[All Fields] OR "fatiguable"[All Fields] OR "fatigue"[MeSH Terms] OR "fatigue"[All Fields] OR "fatigued"[All Fields] OR "fatigues"[All Fields] OR "fatiguing"[All Fields] OR "fatigueability"[All Fields] OR "neuromuscular fatigue"[All Fields] OR "physical fatigue"[All Fields] OR "muscle fatigue"[All Fields] OR "task failure"[All Fields] OR "fatigue resistance"[All Fields] OR "state fatigue"[All Fields] OR ("fatigability"[All Fields] OR "fatigable"[All Fields]) OR "CNS fatigue"[All Fields] OR "central nervous system fatigue"[All Fields] OR "Peripheral fatigue"[All Fields]))

AND

((EMG OR "electromyography" OR "electromyographic activity" OR "muscle activation" OR "neuromuscular activity" OR "motor unit" OR "muscle fiber" OR "muscle contraction" OR "electrical activity" OR "muscle performance" OR "motor control" OR "myoelectric signals" OR "muscle fatigue" OR "electrical stimulation" OR "surface EMG" OR "intramuscular EMG"))

2. WoS:

(("electroencephalography" OR "EEG" OR "neural correlates" OR "neural activity" OR "evoked potentials" OR "ERP" OR "event-related potentials" OR "spectral power" OR "cortical coherence" OR "coherence" OR "frequency bands" OR "brain waves" OR "brainwave" OR "neurophysiological" OR "neurophysiology" OR "brain oscillations")

AND

("fatiguability" OR "fatiguable" OR "fatigue" OR "fatigued" OR "fatigues" OR "fatiguing" OR "fatigueability" OR "neuromuscular fatigue" OR "physical fatigue" OR "muscle fatigue" OR "task failure" OR "fatigue resistance" OR "state fatigue" OR "CNS fatigue" OR "central nervous system fatigue" OR "peripheral fatigue")

AND

("EMG" OR "electromyography" OR "electromyographic activity" OR "muscle activation" OR "neuromuscular activity" OR "motor unit" OR "muscle fiber" OR "muscle contraction" OR "electrical activity" OR "muscle performance" OR "motor control" OR "myoelectric signals" OR "electrical stimulation" OR "surface EMG" OR "intramuscular EMG"))

3. Embase:

('electroencephalography'/exp OR 'eeg' OR 'neural correlates' OR 'neural activity' OR 'evoked potential'/exp OR 'event related potential' OR 'spectral power' OR 'cortical coherence' OR 'coherence' OR 'frequency bands' OR 'brain wave'/exp OR 'brainwave' OR 'neurophysiologic' OR 'neurophysiological' OR 'neurophysiology'/exp OR 'brain oscillation') AND ('fatiguability' OR 'fatiguable' OR 'fatigue'/exp OR 'fatigued' OR 'fatigues' OR 'fatiguing' OR 'fatigueability' OR 'neuromuscular fatigue' OR 'physical fatigue' OR 'muscle fatigue' OR 'task failure' OR 'fatigue resistance' OR 'state fatigue' OR 'cns fatigue' OR 'central nervous system fatigue') AND ('emg' OR 'electromyography'/exp OR 'electromyographic activity' OR 'muscle activation' OR 'neuromuscular activity' OR 'motor unit' OR 'muscle fiber' OR 'muscle contraction' OR 'electrical activity' OR 'muscle performance' OR 'motor control' OR 'myoelectric signals' OR 'muscle fatigue' OR 'electrical stimulation' OR 'surface emg' OR 'intramuscular emg')
